# Resolving binding pathways and solvation thermodynamics of plant hormone receptors

**DOI:** 10.1016/j.jbc.2023.105456

**Published:** 2023-11-08

**Authors:** Chuankai Zhao, Diego E. Kleiman, Diwakar Shukla

**Affiliations:** 1 Department of Chemical and Biomolecular Engineering, University of Illinois at Urbana-Champaign, Urbana, Illinois, USA; 2 Center for Biophysics and Quantitative Biology, University of Illinois at Urbana-Champaign, Urbana, Illinois, USA; 3 Department of Plant Biology, University of Illinois at Urbana-Champaign, Urbana, Illinois, USA; 4 Department of Bioengineering, University of Illinois at Urbana-Champaign, Urbana, Illinois, USA

**Keywords:** plant hormones, hydration site analysis, molecular dynamics, Markov state model, inhomogenous solvation theory

## Abstract

Plant hormones are small molecules that regulate plant growth, development, and responses to biotic and abiotic stresses. They are specifically recognized by the binding site of their receptors. In this work, we resolved the binding pathways for eight classes of phytohormones (auxin, jasmonate, gibberellin, strigolactone, brassinosteroid, cytokinin, salicylic acid, and abscisic acid) to their canonical receptors using extensive molecular dynamics simulations. Furthermore, we investigated the role of water displacement and reorganization at the binding site of the plant receptors through inhomogeneous solvation theory. Our findings predict that displacement of water molecules by phytohormones contributes to free energy of binding *via* entropy gain and is associated with significant free energy barriers for most systems analyzed. Also, our results indicate that displacement of unfavorable water molecules in the binding site can be exploited in rational agrochemical design. Overall, this study uncovers the mechanism of ligand binding and the role of water molecules in plant hormone perception, which creates new avenues for agrochemical design to target plant growth and development.

Plant hormones are small molecules that are naturally produced *in planta* at low concentrations to regulate growth, development, and stress responses (1, 2). There are nine major classes of plant hormones that have been identified, including abscisic acid (ABA), auxin, brassinosteroid (BR), cytokinin, ethylene, giberellin (GA), jasmonic acid (JA), salicylic acid (SA), and strigolactone (SL). Generally, plants adjust their levels of plant hormones in response to a changing environment, and plant hormones act collectively to regulate a variety of responses (1, 2). Over the last decades, exciting progress has been made in understanding different aspects of plant hormone biology, including hormone biosynthesis, transport, perception, and response. In particular, the discovery of plant receptors and their crystal structures (except for ethylene) has stimulated characterization of hormone perception and signal transduction at the molecular level (3, 4, 5, 6, 7, 8, 9, 10, 11, 12, 13, 14, 15, 16). Mechanistic understanding of hormone perception has triggered the development of synthetic agrochemicals for targeting plant receptors, which can be utilized in agricultural control of crop growth and development (17).

Plant hormones are recognized by their receptor proteins through a host of specific protein–ligand interactions. Understanding the driving force of those protein-ligand interactions is crucial for the rational design of new hormone agonists with enhanced affinity and selectivity (18). Water has been increasingly recognized as playing a key role in protein-ligand interactions (19, 20, 21, 22). Structurally, water molecules can be classified into four categories in regard to their role in ligand binding: bulk, buried, interface, and surface (23). Bulk waters correspond to the surrounding solvent, whose residence time around the solute is extremely short (in the order of picoseconds). Buried waters exist within protein cavities and they are isolated from the bulk solvent, which results in extremely long residence times (in the order of milliseconds). Interfacial waters can bridge interactions between proteins and ligands and have varying residence times. Lastly, surface waters represent the hydration shells of the ligand and protein. Surface waters present varying residence times, but usually, they exchange much faster than interfacial waters. Of these categories, it is surface waters that are typically affected by a binding event. Upon ligand binding, water molecules in the cavity may be displaced, replaced, or retained to accommodate protein-ligand interactions. All these changes can affect the binding thermodynamics due to enthalpic or entropic effects.

Due to the extremely dynamic nature of water molecules, it remains challenging to experimentally characterize the structural and thermodynamic solvation properties of the binding cavity in a protein (20, 21, 23). For this reason, many computational techniques which operate at different levels of theory have been proposed to model waters at protein-ligand interfaces (24). Some methods provide structural information only, such as knowledge– and interaction–based site prediction techniques. Knowledge–based site prediction methods utilize high-quality experimental information to learn common structural patterns associated with hydration sites. Interaction–based site prediction employs a spatial search and an interaction model to determine favorable water sites. Other methods are able to simultaneously provide the positions of hydration sites and their thermodynamic properties. Inhomogeneous solvation theory (IST) allows us to compute enthalpic and entropic terms of water molecules through the use of explicit solvent molecular dynamics (MD) simulations (25). MD simulations capture the motion of proteins in a solution environment, making it an ideal method for addressing the challenges in studying water in the buried regions of a protein (26). MD simulations have also been used extensively in studying detailed protein-ligand binding mechanisms (27, 28, 29, 30, 31). In recent years, a range of computational tools based on IST have emerged as powerful techniques to investigate solvation structural and thermodynamic properties of protein binding cavities. Based on these tools, solvation thermodynamic information can be exploited in predicting ligand binding affinity and rational ligand optimization (25, 32, 33).

In this work, we performed large-scale MD simulations (aggregate *∼*786 *μ*s) to investigate the role of water on the perception of eight major classes of plant hormones (auxin, JA, GA, SL, BR, cytokinin, SA, and ABA) by their receptors in *Arabidopsis thaliana* (Fig. 1 and see Table S1 for quality metrics on Protein Data Bank (PDB) structures used in MD simulations) (30, 34, 35, 36, 37). Phytohormone receptors are generally conserved across plant taxa and especially among angiosperms (38), which makes the study of *A. thaliana* receptors useful to understand the molecular mechanisms behind phytohormone perception in general. Moreover, several receptors for the same hormone are expressed in the same organism. Due to the high homology within these families, the structures for different receptors align well. In other words, studying a single member of each receptor family is useful to understand the binding mechanism. The ligand-complex systems were chosen to comprise all canonical hormone-receptor pairs (with the exception of ethylene).

**Figure 1:**
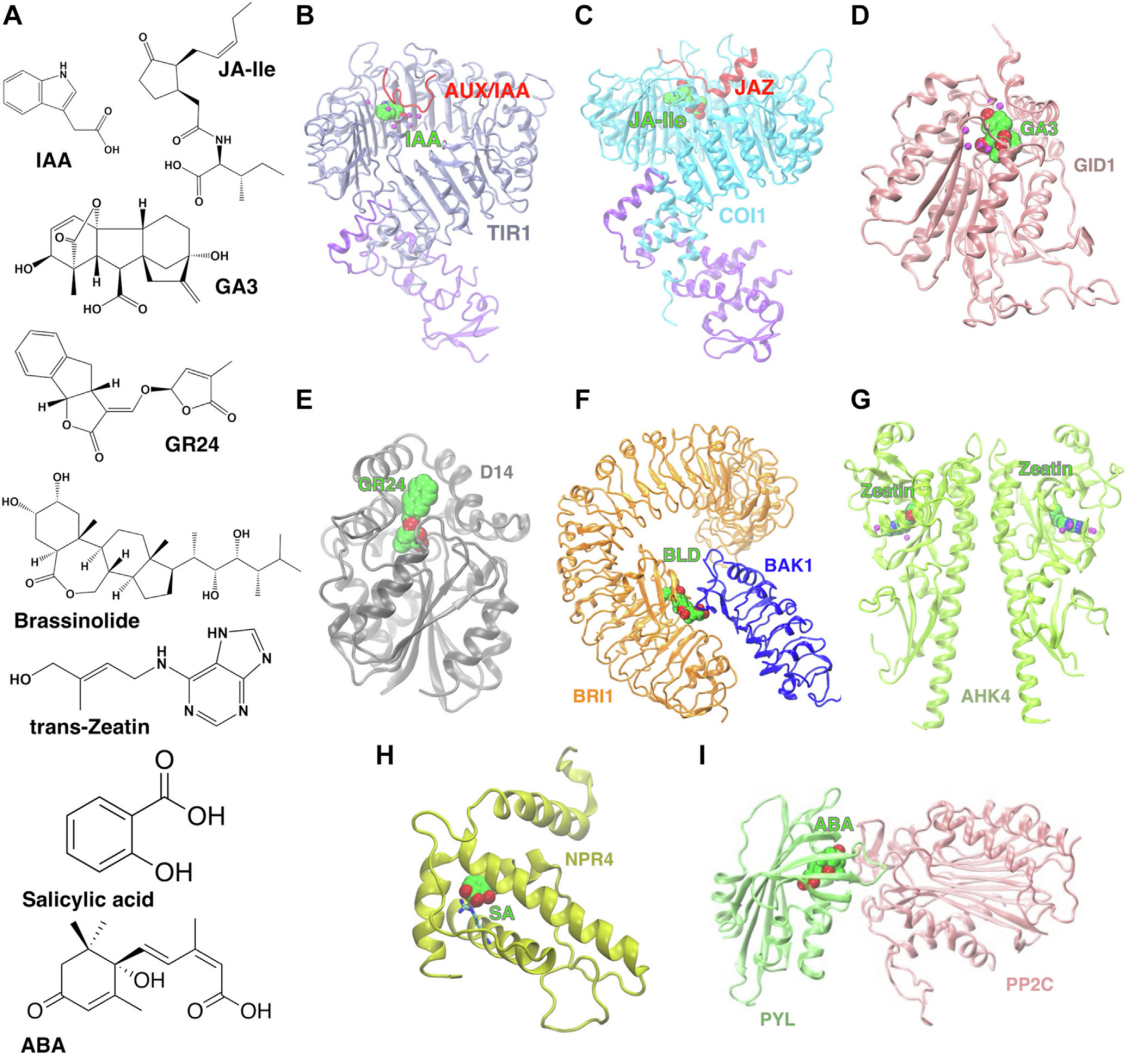
**Plant hormones and their receptors**. *A*, molecular structures of eight major classes of plant hormones. *B*, TIR1-AUX/IAA for auxin (PDB ID: 2P1Q (3)), (*C*) COI1-JAZ for jasmonate (PDB ID: 3OGL (5)), (*D*) GID1 for gibberellin (PDB ID: 2ZSH (4)), (*E*) D14 for strigolactone (PDB ID: 5DJ5 (11)), (*F*) Extracelluar domains of BRI1 and BAK1 for brassinosteroid (PDB ID: 4LSX (62)), (*G*) extracelluar sensor domain of AHK4 for cytokinin (PDB ID: 3T4L (10)), (*H*) NPR4 and salicylic acid (PDB ID: 6WPG (13)), (*I*) PYL2-PP2C and ABA (PDB ID: 4LA7 (63)). ABA, abscisic acid; PDB, Protein Data Bank.

Markov state model (MSM) analysis was used to characterize the thermodynamics associated with the binding processes (39, 40). By analyzing the binding pathways, we assessed the key role of water in plant hormone binding. Furthermore, we characterized the solvation structural and thermodynamic properties of the eight plant receptors by analyzing MD trajectories on *apo* proteins *via* IST-based hydration site analysis (HSA) (Table S2). Based on these data, we analyzed the properties of water molecules upon the binding of plant hormones, and quantified their contribution to overall free energy of binding. Finally, we characterized the thermodynamic properties of unfavorable water molecules that are retained in the bound complexes. We showed that excluding such unfavorable water molecules by optimizing a ligand can lead to enhanced ligand binding affinity. Overall, our findings elucidate the role of water in perception of plant hormones and create potential avenues for agrochemical discovery to target plant receptors.

## Results

### Long timescale MD simulations capture the pathway of plant hormone binding and the relevance of water in this process

First, we sought to characterize how water in the binding sites affect phytohormone binding processes. We performed large timescale MD simulations (aggregate *∼*786 *μ*s) to capture the binding of IAA, JA-Ile, GA3, trans-zeatin, and SA to their respective receptors, followed by MSM analysis on the binding trajectories (Supplementary methods, Fig. S1 and Tables S3–S8). Using MSMs, the pathway for the binding of plant hormones and the associated thermodynamics and kinetics can be quantified (39, 40). We also obtained the data for the binding of brassinolide (BLD), GR24, and ABA from the past studies (BLD from ref. (30), GR24 from ref. (34), and ABA from ref. (35)). We reported the free energy landscapes that describe the overall binding of plant hormones as well as the pathways for these processes identified from transition path theory (TPT) (Figs. S2–S9).

**Figure 2:**
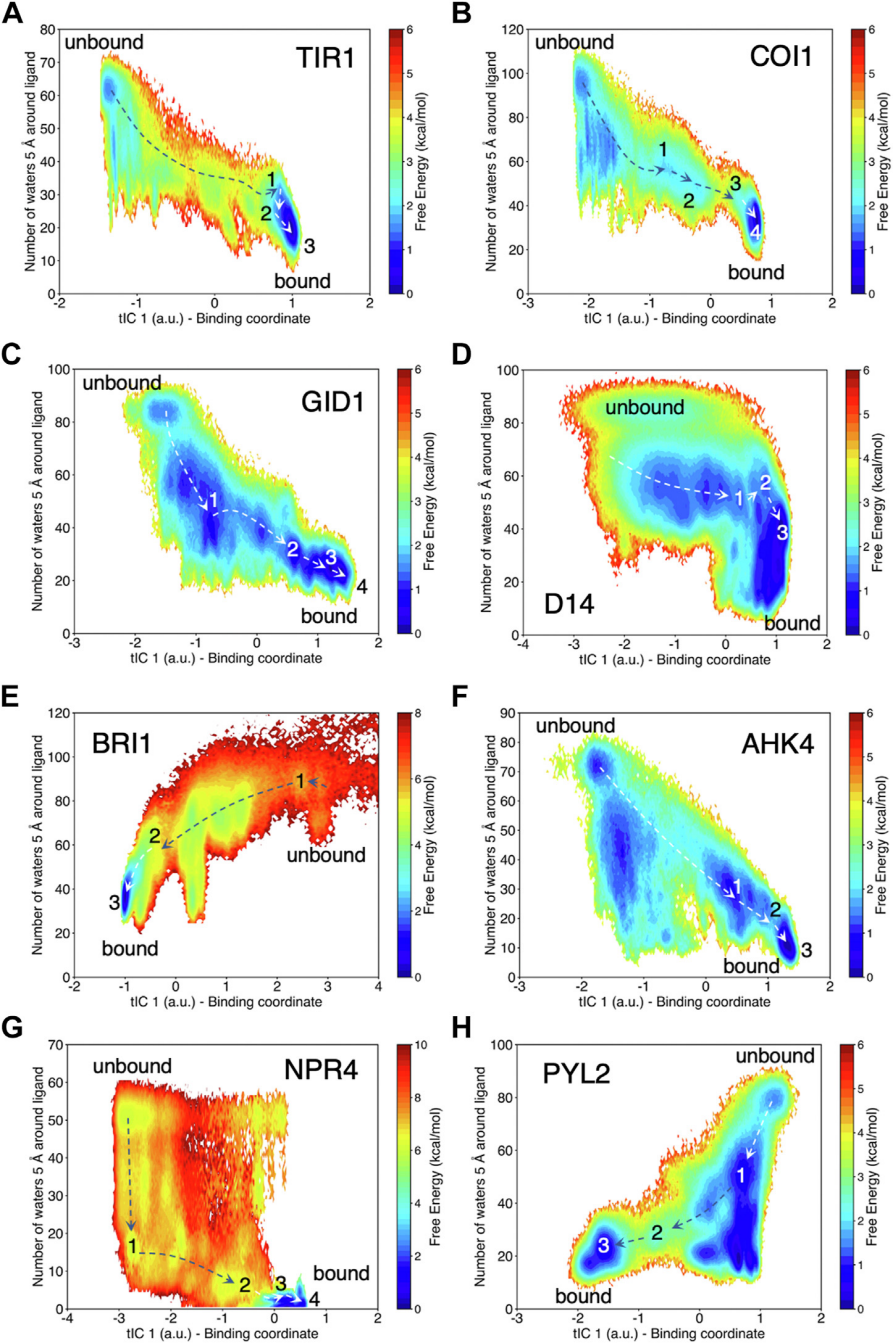
**Free energy landscapes for ligand solvation of plant hormones upon receptor binding**. *A*, IAA binding to TIR1, (*B*) JA-Ile binding to COI1, (*C*) GA3 binding to GID1, (*D*) GR24 binding to D14, (*E*) BLD binding to BRI1, (*F*) trans-zeatin binding to AHK4, (*G*) SA binding to NPR4, and (*H*) ABA binding to PYL2 (Panels are reused in Figs. S2*A*–S9*A*). The x coordinate represents the slowest degree of freedom from time-lagged independent component analysis, which captures the binding process. The y coordinate is the number of water molecules within 5 Å of ligand (approximately first and second solvation shells). ABA, abscisic acid; BLD, brassinolide; BR, brassinosteroid; GA, giberellin; JA, jasmonic acid; SA, salicylic acid.

Employing the resulting MSMs, we interrogated the role of waters in the binding processes. The solvation shell of a phytohormone accounts for the interfacial waters that bridge the pocket-ligand interactions during hormone-receptor complexation. These solvent molecules must be either displaced or reorganized to accommodate the ligand-protein interactions. These processes can pose significant energy barriers (2–8 kcal/mol) for ligand binding. For this reason, we assessed the role of water molecules in hormone binding by computing the free energy landscape of ligand hydration against the binding coordinate resolved by time-independent component analysis (Fig. 2). These plots show that the binding process is correlated with ligand desolvation. The presence of a free energy barrier along the solvation coordinate shows that water displacement is a key process for hormone-receptor complexation. Due to the diversity of the ligand-receptor systems analyzed, stark contrasts are also evidenced in these free energy landscapes. For example, the plot for SA-NPR4 shows a funnel-like landscape with a large energy barrier (*∼*8 kcal/mol) between states 1 and 2. As the binding process of SA continues, the ligand loses all the waters in its hydration shell, mostly due to the formation of hydrophobic interactions with pocket residues. On the other hand, systems such as GA3-GID1, which present several bridging waters, show lower energy barriers (*∼*2 kcal/mol) along the binding pathway. Unlike other systems, GR24-D14 shows relatively small (*<*1 kcal/mol) barriers associated with water displacement. It is also observed that some of the systems (*e.g.*, trans-Zeatin-AHK4 and ABA-PYL2) show free energy minima that are not associated with the main binding pathway and indicate alternative ligand desolvation paths. Such minima are generally associated with off-pocket binding. For D14 and BRI1, the time-independent component analysis plots in Figs. S5*C* and S6*C* show additional minima that are not related to binding (Figs. S27 and S28).

Although free energy landscapes allow us to assess the relevance of the solvent in the binding process in terms of the water displacement barrier, several important factors remain unaccounted for by this methodology. Namely, the distribution of hydration clusters in the binding site and their thermodynamic properties need to be evaluated individually to determine if their displacement would further stabilize the bound complex. This is the focus of the following sections.

### Phytohormone receptors display diverse structural and thermodynamic solvation properties

**Figure 3:**
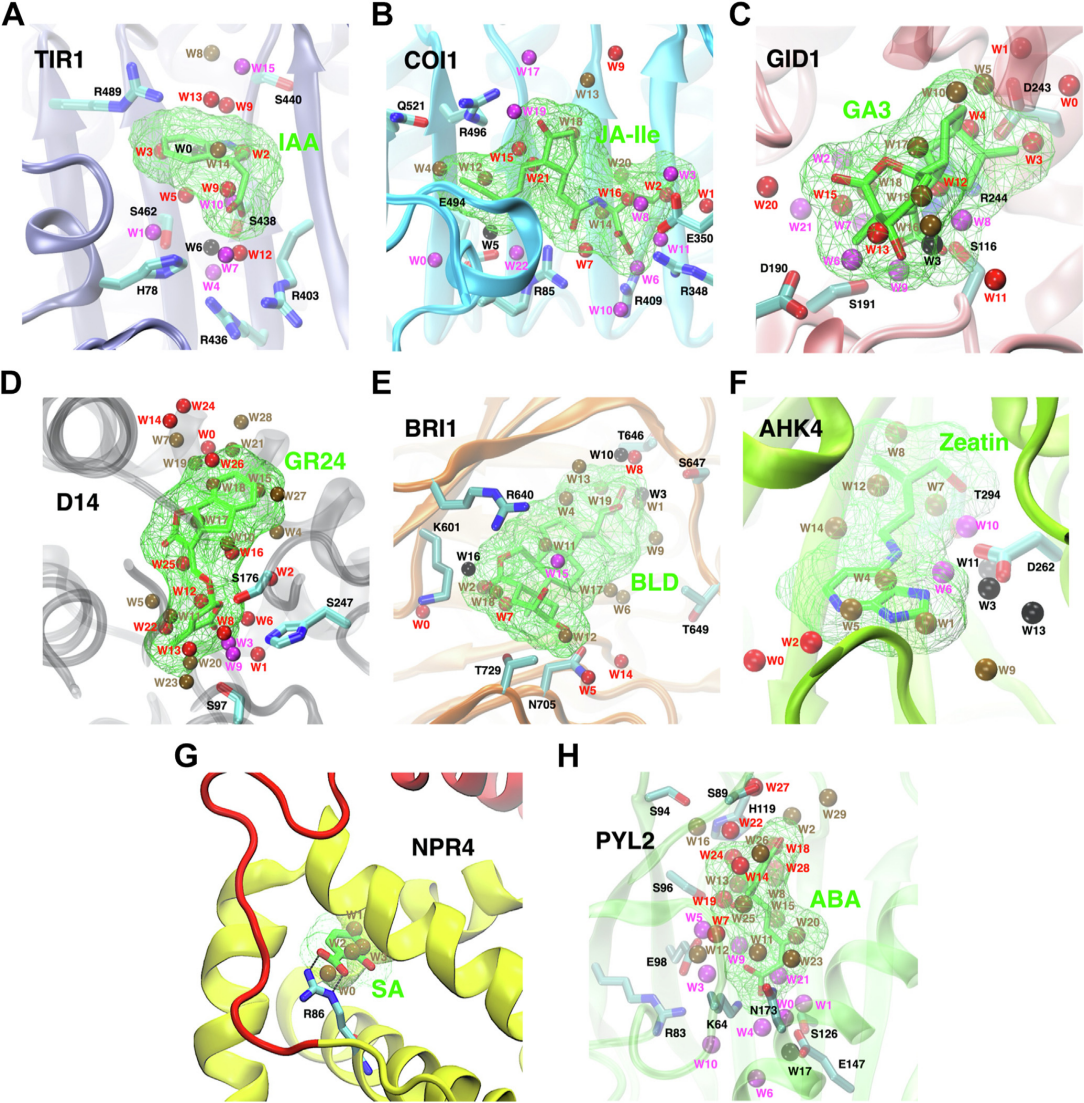
**Visualization of hydration sites at the binding sitesofplant hormone receptors.***A,* TIR1 in *iceblue*, (*B*) COI1 in *cyan*, (*C*) GID1 in *pink*, (*D*) D14 in *gray*, (*E*) BRI1 in *orange*, (*F*) AHK4 in *lime*, (*G*) NPR4 in *yellow/red*, and (*H*) PYL2 in *green*. The plant hormones in the bound poses and their envelopes (probe radius of 1.5 Å) are shown in *green*. The polar and charged residues at the binding sites are shown in *cyan*. Hydration sites are numbered based on their occupancy and colored according to their energetic states: En.F (*red*), En.U (*ochre*), Fr.F (*magenta*), and Fr.U (*black*). BR, brassinosteroid.

In this section, we focused on characterizing the structural and thermodynamic properties of hydration sites in the hormone binding pockets to resolve the detailed energetic contributions of waters in ligand-receptor complexation. In order to investigate the solvation pattern of the binding site of *apo* plant receptors, we performed 100 ns explicit solvent MD simulations on the eight receptors. The crystal structures of the plant receptors in the hormone-bound conformations were used and conformational restraints on protein backbone were applied in MD simulations (Experimental procedures). IST-based HSA was then used to analyze the trajectories from MD simulations in order to characterize solvation structural and thermodynamic properties of the binding cavities (25, 32, 33). Hydration sites define the centers in the binding pocket where water resides with the highest probabilities. Using IST, the enthalpic (including both protein-water and water-water interaction energy, denoted as *E*_*sw*_ and *E*_*ww*_) and entropic properties (excess entropy relative to bulk water *−TS*^*e*^) of water molecules in hydration sites can be estimated. Furthermore, structural properties of water molecules such as the number of hydrogen bonds with protein (*N*^*HB*^) and neighboring water molecules (*N*^*HB*^) can be determined. We identified the hydration sites in the eight plant receptors, which are numbered according to water occupancy probabilities (*f*_*o*_) (Fig. 3 and Tables S9–S16). Based on the local environment, the hydration sites were classified into apolar (A), polar (P), and charged (C) sites. In addition, the sites were classified into either favorable (F) or unfavorable (U) by comparing *E*_*tot*_ with bulk water. Lastly, these sites were also classified into either enhanced (En, favorable water-water interactions) and frustrated (Fr, unfavorable water-water interactions) by comparing *E*_*ww*_ of each site and bulk water (Experimental procedures and Fig. S10).

In total, we have identified 159 hydration sites in the eight receptors (A: 22.64%, P: 44.03%, C: 33.33%, Table 1). For five of the receptors (TIR1, GID1, BRI1, AHK4, and PYL2), the presence of crystallographic waters in the X-ray structure permits the comparison between predicted water sites and experimentally resolved ones. The computed locations of the hydration sites match (up to 2 Å) with the water locations reported in the hormone-bound crystal structures in 50%, 100%, 100%, 100%, and 64% of the cases respectively (Fig. S11). The limit of 2 Å was selected based on the resolution of the technique, which detects hydration sites with a radius of 1 Å (22). Consequently, a distance of less than 2 Å between the computed site and the experimental one indicates that the two sites clash and therefore are counted as the same molecule. Only waters located up to 5 Å away from the ligand in its bound pose were considered. It must be noted that the structure used for PYL2 (PDB ID: 3KDH) does not contain the ligand, so the MD-resolved bound position was used. For COI1 (PDB ID: 3OGK), no crystallographic waters were resolved. For NPR4 (PDB ID: 6WPG) and D14 (PDB ID: 5DJ5), no crystallographic waters were present in the binding pocket, although other hydration sites were resolved. The match between theoretical and experimentally detected water sites shows that unbiased MD simulations were both able to capture ligand binding and hydration clusters accurately, demonstrating the maturity of solvent analysis methods (24).

**Table 1:** The average thermodynamic and structural quantities for all the hydration sites identified from the binding cavity of plant hormone receptors (energy unit: kcal/mol)

Type	Count	$f_{o}$	$E_{\mathrm{sw}}$	$E_{\mathrm{ww}}$	$E_{\mathrm{tot}}$	$E_{\mathrm{ww}}^{\mathrm{nbr}}$	$N_{\mathrm{nbr}}$	$f_{\mathrm{enc}}$	$N_{\mathrm{sw}}^{\mathrm{HB}}$	$N_{\mathrm{ww}}^{\mathrm{HB}}$	$f_{\mathrm{ww}}^{\mathrm{HB}}$	$N_{\mathrm{ww,lost}}^{\mathrm{HB}}$	frac.
pure water	-	-	0.00	−9.53	−9.53	−1.36	5.26	0.00	0.00	3.33	0.63	0.00	-
A.En.F	11	0.40	−2.15	−7.92	−10.1	−1.63	4.21	0.20	0.01	3.17	0.75	0.16	0.64
A.En.U	24	0.39	−1.97	−7.01	−8.98	−1.72	3.46	0.34	0.01	2.62	0.76	0.71	0.67
A.Fr.U	1	0.30	0.16	−5.45	−5.29	−1.10	4.00	0.24	0.07	2.35	0.59	0.98	0.00
C.En.F	11	0.61	−6.06	−4.56	−10.6	−1.64	2.64	0.50	1.20	2.00	0.76	1.33	0.36
C.En.U	11	0.65	−4.01	−4.67	−8.68	−1.86	2.23	0.57	0.89	1.69	0.75	1.66	0.73
C.Fr.F	26	0.64	−9.19	−1.78	−11.0	−0.83	2.65	0.50	1.47	1.55	0.58	1.78	0.20
C.Fr.U	5	0.53	−7.23	−1.89	−9.12	−0.96	2.60	0.51	1.01	1.54	0.64	1.79	0.40
P.En.F	29	0.52	−4.64	−5.49	−10.1	−1.69	2.83	0.46	0.95	2.14	0.76	1.19	0.38
P.En.U	28	0.42	−3.20	−5.58	−8.78	−1.75	2.70	0.49	0.64	1.99	0.75	1.36	0.50
P.Fr.F	8	0.47	−6.25	−4.22	−10.5	−1.15	3.14	0.40	1.10	2.00	0.64	1.33	0.38
P.Fr.U	5	0.48	−5.06	−4.13	−9.19	−1.22	2.82	0.46	1.15	1.72	0.60	1.61	0.60
Undisplaced	86	0.53	−5.52	−4.40	−9.93	−1.37	2.88	0.46	0.97	1.97	0.69	1.36	-
Displaced	73	0.48	−3.86	−5.60	−9.46	−1.65	2.99	0.43	0.60	2.21	0.75	1.13	-

Data for pure water is included for comparison.

**Figure 4:**
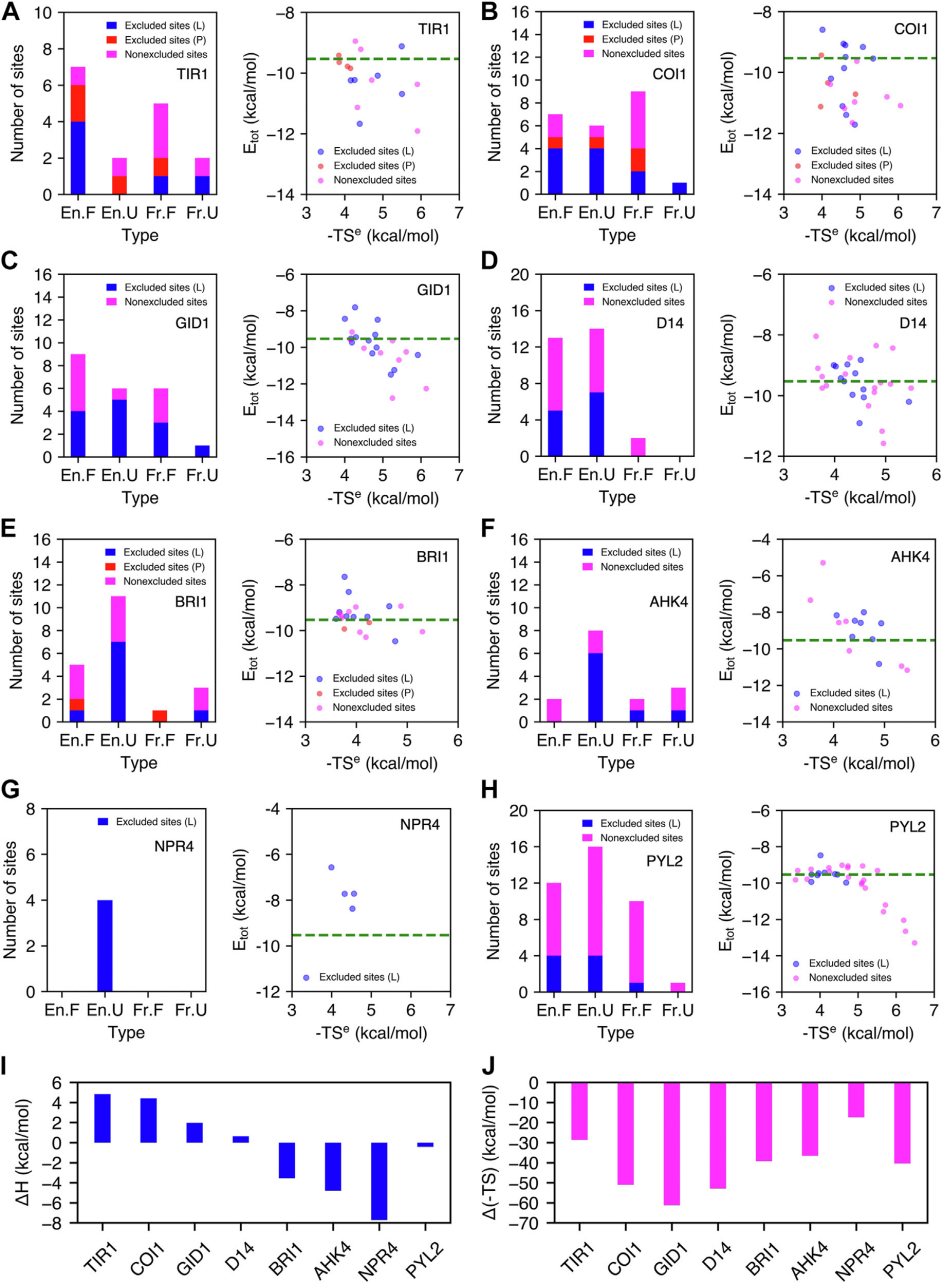
**Displacement of water from hydration sites at the binding cavities**. (*Left*) number of hydration sites (En.F, En.U, Fr.F, and Fr.U) that are displaced upon the binding of plant hormones and coreceptors, and (*right*) Etot and -TSe of the hydration sites for (*A*) TIR1, (*B*) COI1, (*C*) GID1, (*D*) D14, (*E*) BRI1, (*F*) AHK4, (*G*) NPR4, and (*H*) PYL2. *Green dashed line* corresponds to Etot for bulk water. The hydration sites displaced by plant hormones (L) and coreceptors (P) are shown in *blue* and *red*, and the retained sites are shown in *magenta*. Overall (*I*) enthalpic and (*J*) entropic contribution of water displacement to free energy of binding of plant hormones to the eight receptors. (*Right panels* of *A*–*H* are reused in Fig. S10).

For all the eight receptors, there are generally more enhanced sites than frustrated sites (Fig. 4). For TIR1, COI1, GID1, and PYL2, due to the presence of several charged residues at the binding site, there are enhanced populations of frustrated sites and frustrated sites are generally favorable (Fig. 4, *A*–*C*, and *H*). In other words, the water molecules are stabilized by the charged residues (so the total energy is favorable) but this disrupts the orientation of the solvent, which prevents the formation of H-bonds across neighboring waters (so these waters are frustrated). In contrast, the hydration sites in D14, BRI1, AHK4, and NPR4 are dominantly enhanced due to overall hydrophobic environment in the binding cavity (Figs. 3, *D*–*G* and 4, *D*–*G*). In addition, the number of En.U sites is greater than any other type of hydration site for D14, BRI1, AHK4, NPR4, and PYL2 (Fig. 4, *D*–*H*). Overall, MD simulations and HSA have provided detailed characterization of solvation properties at the binding site of plant receptors.

### Displaced water molecules from enhanced hydration sites contribute to binding affinity *via* entropy gain

We further sought to understand how water molecules in the pocket affect the binding of plant hormones. In this work, we have focused our analysis on one member of endogenous or synthetic analogs of natural plant hormones (IAA for auxin, JA-Ile for JA, GA3 for GA, synthetic analog of SL (GR24), BLD for BR, trans-zeatin for cytokinins, SA, and ABA; Fig. 1*A*). We analyzed the displacement of water molecules by the binding of these ligands, by assuming that any water in the hydration sites that are within 1.5 Å of their bound poses would be excluded. In this way, we identified all the hydration sites to be displaced upon the binding of plant hormones, the functional groups in the ligands responsible for displacing certain types of hydration sites (Fig. 3), and their contributions to the free energy of binding (Fig. 4). To show the exclusion of water molecules along the ligand binding processes, we showed *apo* hydration sites over the binding intermediate states captured from our MD simulations and MSM analysis (Figs. S2–S9). Generally, exclusion of favorable hydration sites would lead to a larger free energy barrier to ligand binding.

Overall, 73 out of 159 hydration sites in all the eight receptors are displaced upon the binding of plant hormones. Sixty displaced sites belong to En categories (82.20%) and 43 sites belong to U categories (58.90%). Generally, water molecules in enhanced and unfavorable hydration sites are more likely to be displaced upon the binding of plant hormones. The fractions of displaced hydration sites in apolar, polar, and charged sites are 64.22%, 44.29%, and 36.21% respectively. The displaced hydration sites tend to have relatively weaker protein-water interaction compared to the undisplaced sites (Table 1).

The enthalpic contribution of displaced water molecules to overall free energy of binding depends on thermodynamic properties of water molecules. Other than TIR1, the majority of water molecules displaced by the ligands are from En.U hydration sites. Displacing En.U water molecules leads to a favorable contribution to enthalpy change in binding, in addition to their favorable entropic contribution. En.U hydration sites are generally displaced by a hydrophobic alkyl chain or a bulky ring of the ligands (Fig. 3, *B*–*H*). Strikingly, seven out of nine displaced hydration sites in BRI1, six out of eight displaced hydration sites in AHK4, and all four displaced sites in NPR4 are En.U (Fig. 4, *E*–*G*). The displacement of one Fr.U hydration site in BRI1 and AHK4 further leads to negative binding enthalpy changes. Overall, expelling water molecules from the binding site of BRI1 and AHK4 contribute to free energy of binding both enthalpically and entropically (Fig. 4, *I* and *J*). The second major type of displaced hydration site is En.F site. There are 4 to 5 water molecules from En.F sites being expelled into the bulk for TIR1, COI1, GID1, D14, and PYL2 (Fig. 4, *A*–*E*). Displacement of such favorable sites result in enthalpic penalty for ligand binding, which requires to be compensated by favorable protein-ligand interactions. Generally, En.F sites are displaced by a bulky ring or a polar functional group in the ligands (Fig. 3, *A*–*E* and *H*).

Fr.F is the third type of hydration site being displaced as seen in TIR1, COI1, GID1, AHK4, and PYL2. Fr.F is usually observed around the charged residues in the binding cavity (Fig. 3, *A*–*F* and *H*). Displacing Fr.F site can result in significant enthalpic penalty to ligand binding, which requires to be compensated by strong protein-ligand interactions. A common observation is that Fr.F sites are displaced by the carboxylate group of the ligands (Fig. 3, *A*–*C* and *H*) which form electrostatic interactions with charged or polar residues. Taken altogether, displaced water molecules are mostly from En.U hydration sites, and contribute to free energy of binding *via* gain of entropy.

### Identification of unfavorable water molecules in the bound complex for agrochemical optimization

**Figure 5:**
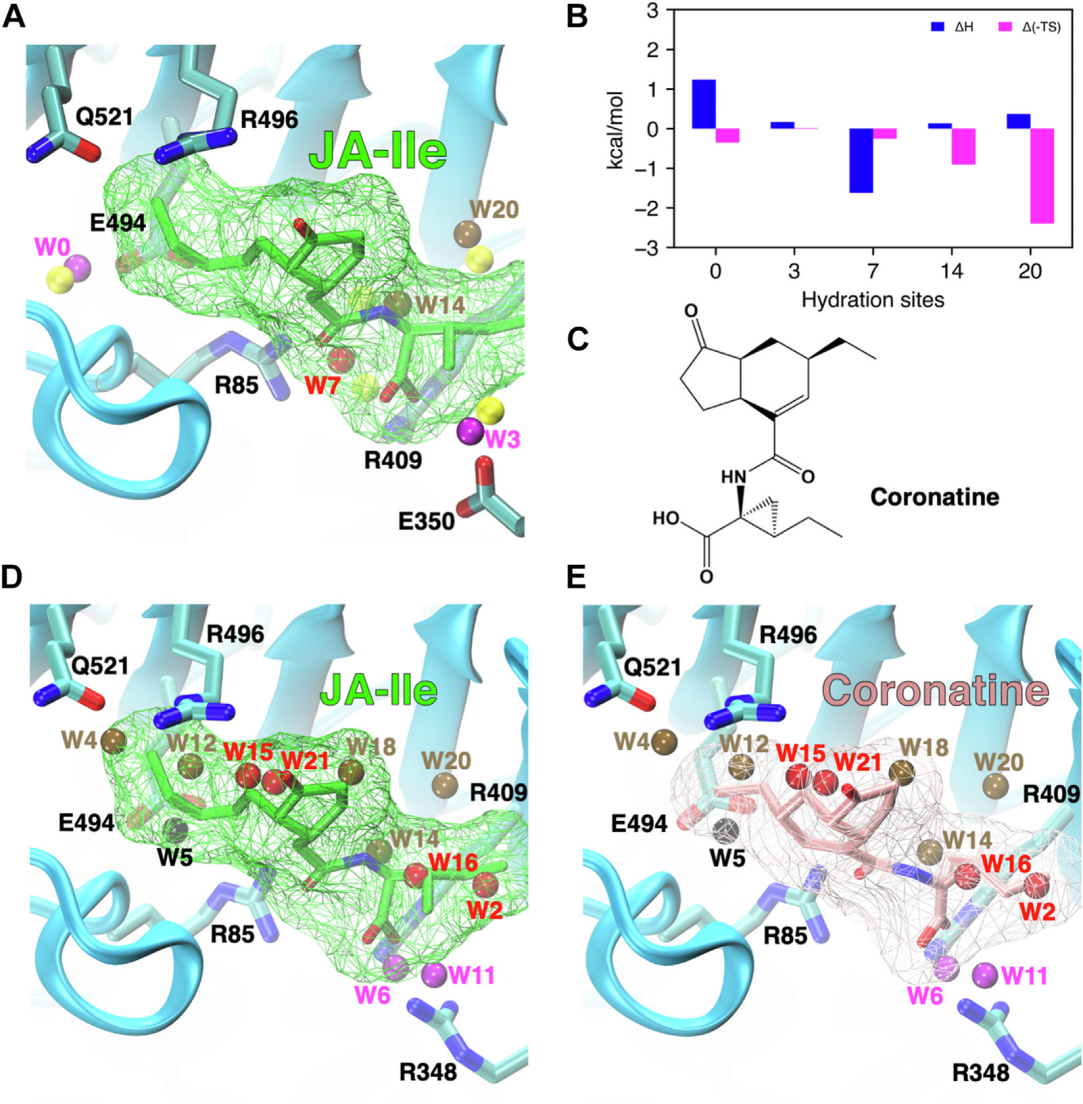
**Molecular origin of enhanced affinity of JA analog coronatine**. *A*, overlay of the conserved apo and holo hydration sites in the binding site of COI1 upon the binding of JA-Ile, and (*B*) the changes in enthalpy and entropy of conserved hydration sites. *C*, molecular structure of coronatine. *D* and *E*, comparison of the exclusion of water molecules upon the binding of (*D*) JA-Ile and (*E*) coronatine to COI1. JA, jasmonic acid.

After the binding of plant hormones, water molecules from some *apo* hydration sites, including unfavorable hydration sites (En.U or Fr.U), may remain in the bound complex (Fig. 4). These water molecules can either be stabilized or destabilized by the presence of plant hormones in the binding site. If a water molecule with unfavorable *E*_*tot*_ exists in the bound complex, optimizing the ligand to exclude such water upon binding can lead to further enthalpy gain for ligand binding. We further sought to determine the thermodynamic properties of water molecules in the bound complex. For each receptor, we extracted the ligand-bound conformation with water molecules present in the binding site from our binding MD simulations. Then, we performed 100 ns MD simulations starting from these snapshots followed by HSA to determine the hydration sites in the bound complex (denoted as *holo*). We note that the *holo* hydration sites may not reproduce all the nonexcluded *apo* hydration sites, since the pocket environment in the chosen bound configuration may deviate from the receptor conformation in *apo* simulations. We therefore focused on characterizing the changes in enthalpy and entropy for the conserved sites captured in *apo* and *holo* simulations, particularly for *apo* unfavorable hydration sites (Figs. 5, *A* and *B* and S12–S17). Note that changes in entropy are expressed as Δ(*−TS*); this is not the conventional *TdS*. While some of the unfavorable *apo* hydration sites are indeed stabilized upon the binding of plant hormones, we identified a few buried unfavorable hydration sites in COI1 (site 14 and 20, Fig. 5, *A* and *B*), D14 (site 5, Fig. S14), and PYL2 (site 2, Fig. S17) that are further destabilized by the bound ligands.

Changes in the number of water-water and water-solute hydrogen bonds upon ligand binding were also plotted (Figs. S19–S25), but it must be noted that all enthalpic interactions are already considered when computing the thermodynamic properties of the waters. We can observe that the presence of the ligand does not change the average number of hydrogen bonds in a predictable manner. In some cases, the ligand disrupts the water-water hydrogen bonds, but these interactions are offset by water-solute hydrogen bonds (*e.g.*, site 5 in TIR1, Fig. S19). The converse can also occur (*e.g.*, site 7 in TIR1, Fig. S19). In other cases, the net number of hydrogen bonds changes, either positively or negatively, but this can be either due to changes in water-water or water-solute hydrogen bonds. Interestingly, there are cases where water-water hydrogen bonds are favored upon ligand binding (*e.g.*, site 4 in PYL2, Fig. S25).

The presence of the unfavorable hydration site 14 in the bound complex of COI1 and JA-Ile can partially explain the 10-fold enhanced affinity of JA analog coronatine relative to JA-Ile (5). Based on the crystal structure of the coronatine-bound complex, the cyclopropane ring present in coronatine would completely exclude the unfavorable hydration site 14, thereby contributing to the overall binding affinity by a predicted value of almost 1 kcal/mol. In addition, the cyclohexene ring results in enhanced hydrophobic interaction between COI1 and the ligand to compensate for the enthalpy loss due to the displacement of favorable hydration sites 15 and 21 (Fig. 5*E*). However, the shorter alkyl chain in coronatine leaves the unfavorable hydration site 4 in the bound complex, which is displaced by JA-Ile. For both JA-Ile and coronatine, unfavorable site 20 remains in the bound complex, which can potentially be excluded by further ligand optimization to enhance binding affinity.

Moreover, HSA could partially explain why the PYL2 antagonist pyrabactin (Fig. S18) binds PYL2 with a slightly lower affinity compared to ABA (41). Based on the crystal structure of the PYL2-pyrabactin complex (PDB ID: 3NJ0 (42)) the sulfonamide group in pyrabactin would displace hydration site 9 (Fr.F), unlike ABA (Fig. S18). This represents an enthalpic penalty which is not compensated by a protein-ligand interaction. Additionally, pyrabactin would not displace waters in sites 13 (En.U), 18, and 24 (both En.F). The retention of favorable sites provides an enthalpic benefit to pyrabactin binding, while the retention of site 13 represents an entropic loss. On the other hand, the pyridyl group in pyrabactin displaces hydration site 15 (En.U) and establishes hydrophobic interactions with nearby residues, which enhances its binding affinity. Also, pyrabactin would not displace site 21 (Fr.F), but in the case of ABA, the carboxylate group displaces this water to form a strong interaction with LYS64. Neither ABA nor pyrabactin displace site 2, which could potentially be excluded through ligand optimization and lead to further complex stabilization.

Lastly, it must be noted that solvation displacement does not necessarily differ between chemically different ligands. Several types of gibberellins exist. In this study, we employed GA3 as the ligand to GID1, but the binding pose of GA4 has also been resolved (4). Both hormones align almost perfectly in the pocket and the displaced waters match exactly. Therefore, HSA predicts no difference in the binding energetics between these two ligands. This is consistent with experimental observations that found no differences among isothermal titration calorimetry curves for GA3, GA4, GA1, and GA7 with *Oryza sativa* GID1 (Fig. S4 in ref. (43)). Overall, these results speculate how displacement of unfavorable water molecules in the binding site of plant receptors may further enhance ligand binding affinity. Moreover, the results also suggest a pathway to agrochemical design by optimizing ligands to exclude certain hydration sites.

## Discussion

In this study, we present an in-depth computational investigation of the role of water on the binding of eight major classes of plant hormones to their receptors. The hydrophobic pocket results in overall enhanced local water structure in the cavity, and water molecules may be either favorable or unfavorable relative to bulk which depends on protein-water and water-water interactions. The presence of charged residues in the pocket mostly leads to frustrated and favorable local water structure. We predict that the binding of plant hormones mostly displace enhanced hydration sites into the bulk, and displaced hydration sites tend to have weaker protein-water interactions. We have also identified the functional groups that are responsible for displacing different types of hydration sites. En.U sites are generally displaced by hydrophobic groups in the hormones, and En.F sites are displaced by bulky hydrophobic groups such as aromatic rings. On the other hand, Fr.F sites are preferably excluded by charged or polar functional groups in the ligand which can form favorable interaction with protein to compensate for the enthalpy loss of favorable water molecules.

Our results predict that displacement and reorganization of water molecules upon the binding of plant hormones have a major contribution to overall free energy of binding. For BRI1, AHK4, and NPR4, the displacement of unfavorable water molecules lead to enthalpy gain of hormone binding. For the rest of the plant receptors, the displacement of water molecules results in some enthalpy loss and therefore unfavorable enthalpic contribution to free energy of binding, due to the exclusion of favorable water molecules. The water molecules in the binding cavity have considerable entropy loss than bulk water. Overall, the displacement of water molecules contribute to free energy of binding *via* gain of entropy of water molecules. Solvation structural and thermodynamic properties of the binding cavity of plant receptors may be exploited in future agrochemical discovery and optimization. We have shown that water molecules from unfavorable hydration sites can remain in the ligand-bound complex, and some of them can be further destabilized by the binding of plant hormones. Enhanced binding affinity can be achieved by redesigning a ligand to displace such unfavorable water molecules. We have utilized the solvation thermodynamic data to explain the enhanced affinity of JA analog coronatine relative to JA-Ile. We have also used these results to rationalize the lower affinity of pyrabactin to PYL2 when compared to ABA. Recent studies have also demonstrated that manipulation of water network in the receptor binding site during lead optimization can result in significant increase of ligand binding affinity (19, 20, 21). Also, methods are being developed to incorporate solvation thermodynamic information into binding affinity evaluation in a virtual screening process (44).

In conclusion, our results provide new insights into the fundamental mechanism of plant hormone perception and suggest that water plays an important role in the binding of plant hormones. We expect that our findings can help advance our understanding of the role of water in protein-ligand binding, and create new avenues for designing hormone agonists with enhanced affinity.

## Experimental procedures

### Initial structures

Table S2 summarizes the details of the initial structures of plant hormones and their receptors, including TIR1/auxin (IAA), COI1/jasmonic acid (JA-Ile), GID1/gibberellin (GA3), AHK4/cytokinin (trans-zeatin), and NPR4/SA that were used for protein-ligand binding MD simulations. The protein and ligand structures were obtained from their crystal structures available in PDB. For IAA and JA-Ile, their TIR1-ASK and COI1-ASK receptor complexes were used for MD simulations. Missing residues of COI1 were added by using the SWISS-MODEL webserver (45). In both TIR1 and COI1, the leucine-rich repeat domain contains an inositol phosphate cofactor (InsP_6_ in TIR1 and InsP_5_ in COI1). Since InsP_5_ was not fully solved in the crystal structure of COI1, we drew an InsP_5_ molecule and aligned InsP_5_ to the structure of partially solved phosphates in 3OGK to obtain the initial structure of COI1- ASK complex. For SA, the SA-binding core (SBC) of NPR4 was used for the simulations. Missing residues of the NPR4 SBC were modeled using the MODELLER package (46) (version 9.25) and the best structure was chosen based on its discrete optimized protein energy score (47).

### Set up of MD simulations

In order to study the binding of phytohormones to their receptors, we performed long timescale MD simulations to capture protein-ligand binding processes. Initially, we used Packmol (https://m3g.github.io/packmol/download.shtml) (48) to randomly place the phytohormone molecules far away (at least 25 Å) from the binding sites of the plant hormone receptors. The receptor-ligand (also including cofactors InsP_6_ in TIR1 and InsP_5_ in COI1) complexes were then solvated with TIP3P water molecules to mimic solution environment. Appropriate numbers of Na^+^ and Cl^*−*^ ions were added to the system to neutralize the system and meet 150 mM salt concentrations. The Amber ff14SB force field was used for the proteins and the general Amber force field (49) was used for phytohormone molecules and cofactors (InsP_5_ or InsP_6_). The partial charges of phytohormone molecules were derived from Antechamber (https://ambermd.org/AmberTools.php) software using AM1-BCC method, and the partial charges of InsP_5_ and InsP_6_ were approximated from the previous theoretical study (50). The simulation systems were minimized for 10,000 steps and then subjected to a series of heating steps to slowly increase the temperature of these systems to 300 K. Finally, the systems were equilibrated in isothermal-isobaric ensemble (300 K, 1 atm) for 1 ns. The simulations were run in isothermal-isobaric (300 K, 1 atm) ensemble using an integration time step of 2 fs. Periodic boundary condition was applied in all MD simulations. The particle-mesh Ewald method was used to treat the electrostatic interactions, along with a 10 Å cutoff distance for van der Waals interactions (51). The SHAKE algorithm (52) was applied to constrain the length of covalent bonds involving hydrogen atoms.

### Steered MD simulations of GID1 receptor to open up N-terminal helix

The crystal structure of GA3 receptor GID1 was in the active form, where the N-terminal helix closes the binding pocket at top of the binding site and interacts with GA3 (4). In order to obtain the open conformation of GID1 without GA3 bound, we have performed 10 *μ*s enhanced MD simulations to sample the conformational dynamics of GID1 in the absence of GA3 in the binding site. The simulations were performed in Amber 14 with steered MD simulation. We then clustered the conformations according to the N-terminal helix conformation, and selected a series of GID1 conformations to use as the starting structures for unbiased adaptive sampling MD simulations of protein-ligand binding.

### Accelerated MD simulations of NPR4 receptor to open up N-terminal helix

The crystal structure of SA receptor (NPR4) was in its bound conformation where the N-terminal helix closes the binding pocket on top of the binding site. Although there is no direct experimental evidence indicating the binding pathway for SA, it is clear that a conformational change is needed for the ligand to enter or exit due to the fact that the pocket is completely closed in the *holo* structure. In order to obtain the open conformation of NPR4 without a bound ligand, we have performed 2 *μ*s accelerated MD simulations to sample the conformational dynamics of NPR4 until SA escapes the binding pocket. The simulation shows that a movement of the *α*N helix is needed for the ligand to escape. Our results seem to explain deuterium-exchange profile experiments performed on the SBC of NPR4 (Fig. S26 and ref. (13)). The simulations were performed in Amber 18 employing the accelerated MD simulation method. Total and dihedral biasing terms were chosen following recommendations from a previous study (53). We then clustered the conformations according to the protein conformation and ligand distance to the binding pocket, and selected a series of starting structures for unbiased adaptive sampling MD simulations of protein-ligand binding.

### Unbiased adaptive sampling of the binding of plant hormones to their receptors

We used adaptive sampling to efficiently sample the protein-ligand binding and the conformational changes in the receptors. In the first round, the phytohormone molecules were placed far away (*>*25 Å) from their receptors at multiple random configurations. MD simulations were launched from the equilibrated system and run for 60 to 160 ns. Then, a certain number of snapshots with the lowest protein-ligand distances were selected to serve as the starting structures for the next rounds of simulations. In the next round, a small amount of MD simulations were performed starting from these configurations, with the initial velocities randomly assigned according to Boltzmann distribution. These iterative samplings were performed for multiple rounds and stopped until we have captured the protein-ligand binding and had enough simulation data to construct statistical models to describe the thermodynamics and kinetics of protein-ligand binding. Tables S3–S7 summarize the details of adaptive sampling for the IAA, JA-Ile, zeatin, GA3, and SA binding to their receptors, respectively.

### MSM construction and hyperparameter selection

MSM is a powerful analytic technique to investigate large-scale unbiased MD simulation data on protein dynamics. MSMs discretize the protein conformational space into a certain number of individual conformational states and estimate the transition probabilities between these states, thereby allowing for investigation of protein dynamics at the timescale inaccessible *via* any conventional MD simulations. To discretize the protein conformational space, all the protein-ligand conformations collected from MD simulations were characterized by calculating a set of features that can best differentiate these configurations (featurization). In this work, we have calculated a set of distances between the atoms in protein and ligand to illustrate the protein-ligand interactions. Specifically, the CA atoms of the amino acids at the binding site (with the closest heavy-atom distance from the ligand within 5 Å) and heavy atoms in the phytohormones were calculated. For GID1, the residue-residue contacts between the binding site and the N-terminal helix were also calculated to characterize the conformational changes in GID1. For the AHK4, the residue-residue contacts between the binding site and the entry loop were also calculated to characterize the conformational changes in AHK4. For NPR4, the residue-residue distances between all *α*-helices were used to capture the changes in protein conformation.

Dimensionality reduction was then performed for clustering the conformation ensemble from MD simulations into kinetically relevant states. Time-lagged independent component analysis was used to identify several slowest degrees of freedom resulted from linear combination of the original metrics (54). K-means clusterings were then performed on the identified slowest motions to cluster the configurations into a certain number of states. MSMs were then constructed based on the clustering results. The matrix of transition probabilities was determined with a lag time *τ* using maximum likelihood approximation. The optimal *τ* was chosen based on the convergence of the implied timescales of MSMs and the number of clusters as well as the number of time-lagged independent components were optimized *via* cross validation ranked by the variational generalized matrix Rayleigh quotient objective function (Fig. S3) (55). All MSMs were constructed using the MSMBuilder 3.4 (56) and the MSM hyperparameters were optimized using the Osprey (https://github.com/msmbuilder/osprey) software (57). The MD simulation data for BLD binding to BRI1 (58 *μ*s) (30), GR24 binding to D14 (198 *μ*s) (34), and ABA binding to PYL2 (107 *μ*s) (35) were taken from the past studies. The final MSM hyperparameters for the eight protein-ligand systems were summarized in Table S15.

### TPT analysis on plant hormone binding pathways

TPT was applied to calculate the probabilities and fluxes for the pathways between ligand-unbound and ligand-bound states in the protein-ligand binding MSMs (58, 59). The ligand-unbound and the ligand-bound states were chosen according to the distance between protein and ligand based on arbitrarily chosen cutoff values. Given the ligand-unbound and the ligand-bound MSM states for TPT analysis, numerous pathways that connected through the ligand-unbound states and the ligand-bound states were obtained, corresponding to plant hormone binding pathways. We reported the top pathways for these plant hormones with the greatest fluxes. The transition pathways were estimated using the MSMBuilder 3.4 python package (56).

### Molecular dynamics simulations and hydration site analysis of plant receptor solvation structure and thermodynamic properties

Explicit-solvent MD simulations on the *apo* and the *holo* plant hormone receptors, TIR1 (IAA), COI1 (JA-Ile), GID1 (GA3), AHK4 (zeatin), BRI1 (brassinolide), D14 (GR24), NPR4 (SA), and PYL2 (ABA) were performed to characterize the solvation structural and thermodynamic properties of their binding sites using Amber 18. The initial structures used for *apo* MD simulations were summarized in Table S2, and the initial structures used for the *holo* MD simulations were obtained from the binding MD simulations. Since an accelerated MD simulation was used for NPR4, the *holo* SBC crystal structure was employed as a starting point instead of using a simulation frame. The receptors were solvated with TIP3P water molecules, and Na^+^ and Cl^*−*^ ions were added to the system to neutralize the system and meet 150 mM salt concentrations. The systems were minimized for 20,000 steps and further equilibrated in NPT ensemble (1 atm, 300 K) for 1 ns to adjust the sizes of simulation boxes. To sample water densities for the binding sites efficiently, a recently developed Monte Carlo/Molecular Dynamics method (60) was used to equilibrate the system in isothermal-isovolumetric (NVT, 300 K) ensemble for 2500 Monte Carlo/Molecular Dynamics cycles. This method allows the sampling of hydration sites even if they are buried in the protein. Each cycle includes 10,000 MC steps that allow for water exchange between binding site and bulk as well as 1000 MD steps that relax water molecules in the binding sites. All heavy atoms of the receptor proteins were harmonically restrained using a force constant of 100 kcal mol^*−*1^ Å^*−*2^. Production MD simulations were launched from the equilibrated structure and run for 100 ns in NVT with restraints applied to protein backbone using a force constant of 2.5 kcal mol^*−*1^ Å^*−*2^. NVT was used in those production simulations to allow for faster convergence of sampled solvent density (25). The coordinates of MD system were saved every 1 ps, resulting in trajectories with 100,000 frames. Then, the analysis calculations were performed using SSTMap python software (https://github.com/KurtzmanLab/SSTMap, “run_hsa” program) (25). SSTMap calculates the structural and thermodynamic properties of water molecules on the surface of receptor cavity that are represented as a set of high-density hydration sites (33). Briefly, the hydration sites were identified by clustering solvent distribution in the binding site into high-occupancy 1 Å spheres. IST (61) was then used to compute the average system interaction energy and excess entropy terms for water in these hydration sites.

## Data availability

Data supporting the findings of this study are available within the article and its Supplementary Information. The data sets generated and/or analyzed during the current study are available at https://github.com/Zhao_Plant_Hormone_JBC_2023 and https.//uofi.box.com/s/qdnxe4pnugiz6i4kj5c4tpeeu1s7knkw. Supporting information accompanies this paper online.

## Supporting information

This article contains supporting information (13).

## Conflict of interest

The authors declare that they have no conflicts of interest with the contents of this article.
