# Supplementary material for: Resolving binding pathways and solvation thermodynamics of plant hormone receptors
Source: J Biol Chem. 2023 Nov 8;299(12):105456. doi: 10.1016/j.jbc.2023.105456 (PMC10704434; doi:10.1016/j.jbc.2023.105456)
Supplement: Supporting information [file mmc1.pdf]

Supporting Information

Resolving Binding Pathways and Solvation Thermodynamics of Plant Hormone Receptors

Chuankai Zhao[1], **Diego E. Kleiman**[2], and **Diwakar Shukla**[*,1,2,3,4,5]

[1] Department of Chemical and Biomolecular Engineering, [2] Center for Biophysics and Quantitative Biology, [3] Department of Plant Biology, [4] National Center for Supercomputing Applications, [5] Department of Bioengineering, University of Illinois at Urbana-Champaign, IL, 61801, USA. Correspondence and requests for materials should be addressed to D.S. (diwakar@illinois.edu).

Contents

1 **Summary of Molecular Dynamics Simulations** **S-3**

2 **Markov State Models Hyperparameters** **S-10**

3 **Hydration Site Analysis Data** **S-11**

4 **Markov State Models Validation** **S-19**

5 **Binding Pathways** **S-20**

6 **Thermodynamic Properties of Hydration Sites** **S-28**

7 **Comparison of Hydration Site Analysis to Crystal Structures** **S-29**

8 **Comparison of *Holo* and *Apo* Hydration Sites** **S-30**

9 **Comparison of Abscisic Acid to Pyrabactin in PYL2** **S-36**

10 **Hydrogen Bond Analysis** **S-37**

11 **NPR4 Conformational Change Analysis** **S-44**

12 **Additional Energy Minima Analysis** **S-45**

1 Summary of Molecular Dynamics Simulations

Table S1. Quality assessment for PDB structures used in this study.

system	PDB ID	resolution (Å)	rfree	clashscore	ramachandran outliers (%)	sidechain outliers (%)
TIR1-ASK/IAA	2P1Q	1.90	0.243	6	0.1	3.5
COI1-ASK/JA-Ile	3OGK	2.80	0.262	21	2.1	12.7
GID1/GA3	2ZSH	1.80	0.226	7	0	1.8
AHK4/Zeatin	3T4L	1.53	0.204	7	0	1.1
NPR4/SA	6WPG	2.28	0.224	13	0	1
D14/GR24	5DJ5	2.40	0.225	4	0	0.5
PYL2/ABA	3KDH	1.65	0.208	7	0.9	1.0
BRI1/BLD	3RGZ	2.28	0.218	6	0.1	0.9

Table S2. Overview of the computational simulations and analysis method performed in this study. For receptor-ligand binding simulations, multiple parallel simulations were performed and their number of atoms and box sizes varied. The numbers provided in this table are average system size.

**simulation**	**starting structure (PDB ID)**	**method**	**force field**	**software**	$\#_{atoms}$	**size (Å^3)**	**ensemble**	**simulation time**
TIR1-ASK/IAA binding	2P1Q	adaptive sampling, MD, MSM	Amber ff14SB, GAFF	Amber 14	~88,000	~95*106*103	NPT	~27.4 μs
COI1-ASK/JA-Ile binding	3OGK	adaptive sampling, MD, MSM	Amber ff14SB, GAFF	Amber 14	~91,000	~93*114*103	NPT	~63.2 μs
GID1/GA3 binding	2ZSH	adaptive sampling, MD, MSM	Amber ff14SB, GAFF	Amber 14	~85,000	~109*95*100	NPT	~129.5 μs
AHK4/Zeatin binding	3T4L	adaptive sampling, MD, MSM	Amber ff14SB, GAFF	Amber 14	~80,000	~78*107*91	NPT	~36.7 μs
NPR4/SA binding	6WPG	adaptive sampling, MD, MSM	Amber ff14SB, GAFF	Amber 18	~50,000	~90*90*90	NPT	~224.0 μs
TIR1 solvation	2P1Q	MD, hydration site analysis	Amber ff14SB	Amber 18	84,725	93*107*102	NVT	100*2 ns
COI1 solvation	3OGK	MD, hydration site analysis	Amber ff14SB	Amber 18	90,751	93*114*103	NVT	100*2 ns
GID1 solvation	2ZSH	MD, hydration site analysis	Amber ff14SB	Amber 18	43,796	85*72*88	NVT	100*2 ns
D14 solvation	4IH4	MD, hydration site analysis	Amber ff14SB	Amber 18	32,233	74*73*75	NVT	100*2 ns
AHK4 solvation	3T4L	MD, hydration site analysis	Amber ff14SB	Amber 18	42,701	69*72*108	NVT	100*2 ns
BRI1 solvation	3RGZ	MD, hydration site analysis	Amber ff14SB	Amber 18	52,263	117*83*67	NVT	100*2 ns
NPR4 solvation	6WPG	MD, hydration site analysis	Amber ff14SB	Amber 18	29,002	68*71*61	NVT	100*2 ns
PYL2 solvation	3KDH	MD, hydration site analysis	Amber ff14SB	Amber 18	38,147	72*75*72	NVT	100*2 ns

Table S3. Summary of the adaptive MD simulations of the binding of auxin to TIR1.

Round	Parallel simulations	Simulation time (ns)	Aggregate (μs)
1	100	80	8
2	100	100	10
3	120	60	7.2
4	40	60	2.4
Total simulation time: ~27.4 μs			

Table S4. Summary of the adaptive MD simulations of the binding of jasmonic acid to COI1.

MD simulations starting from the snapshots where the ligand is far away from the binding site			
Round	Parallel simulations	Simulation time (ns)	Aggregate (μs)
1	100	100	10
2	100	100	10
3	100	100	10
4	50	100	5
5	50	100	5
6	50	100	5
7	50	100	5
8	50	80	4
9	50	80	4
MD simulations starting from the crystal structure where the ligand is bound to COI1			
1	20	100	2
2	50	100	5
Total simulation time: ~63.2 μs			

Table S5. Summary of the adaptive MD simulations of the binding of cytokinin to AHK4.

Round	Parallel simulations	Simulation time (ns)	Aggregate (μs)
1	100	76	7.6
2	50	120	7
3	100	120	12
4	100	120	12
Total simulation time: ~36.7 μs			

Table S6. Summary of the adaptive MD simulations of the binding of giberellin to GID1.

Round	Parallel simulations	Simulation time (ns)	Aggregate (μs)
1	200	78	15.6
2	60	112	6.7
3	100	112	11.2
4	50	112	5.6
5	100	84	8.4
6	100	112	11.2
7	50	112	5.6
8	100	84	8.4
9	600	100	60
Total simulation time: ~129.5 μs			

Table S7. Summary of the adaptive MD simulations of the binding of salicylic acid to NPR4.

MD simulations starting from the crystal structure where the ligand is bound to NPR4			
Round	Parallel simulations	Simulation time (ns)	Aggregate (μs)
1	520	160	83
MD simulations starting from the snapshots where the ligand is far away from the binding site			
1	539	160	86
2	189	80	15
MD simulations starting from the snapshots obtained by aMD			
1	500	80	40
Total simulation time: ~224 μs			

2 Markov State Models Hyperparameters

Table S8. Hyperparameters for the MSMs constructed for the receptor-ligand binding simulations.

**simulation**	**# tICs**	**lag time (ns)**	**# clusters**	**# MSM states**	**data utilization (%)**
TIR1/IAA binding	6	30	100	100	100
COI1/JA-Ile binding	3	30	100	95	99.2
GID1/GA3 binding	2	30	200	200	100
AHK4/Zeatin binding	2	30	200	200	100
BRI1/BLD binding	6	20	200	200	100
D14/GR24 binding	4	20	325	325	100
NPR4/SAL binding	10	20	500	500	100
PYL2/ABA binding	4	30	300	300	100

3 Hydration Site Analysis Data

Table S9. Calculated thermodynamic data (kcal/mol) and structural quantities for each of the 16 hydration sites in *apo* TIR1 receptor identified by clustering the active-site solvent density distribution. 'Neat' represents the pure TIP3P water.

index	type	f_o	E_{sw}	E_{ww}	E_{tot}	E_{ww}^{nbr}	$-TS^e$	N_{nbr}	f_{enc}	N_{sw}^{HB}	N_{ww}^{HB}	f_{ww}^{HB}	$N_{ww,lost}^{HB}$
neat	-	-	0.00	-9.53	-9.53	-1.36	0.00	5.26	0.00	0.00	3.33	0.63	0.00
0	P.Fr.U	0.88	-5.64	-3.48	-9.12	-1.32	5.49	2.24	0.57	1.40	1.38	0.61	1.95
1	C.Fr.F	0.87	-10.74	-1.17	-11.91	-1.25	5.91	2.34	0.56	1.80	1.57	0.67	1.76
2	P.En.F	0.75	-5.92	-4.17	-10.08	-1.40	4.86	2.49	0.53	1.77	1.61	0.65	1.72
3	P.En.F	0.65	-6.03	-4.66	-10.68	-1.79	5.50	2.33	0.56	1.58	1.78	0.76	1.55
4	C.Fr.F	0.59	-11.42	1.05	-10.37	0.22	5.90	0.83	0.84	2.70	0.33	0.39	3.00
5	P.En.F	0.59	-3.41	-6.82	-10.23	-1.70	4.71	4.01	0.24	0.25	2.90	0.72	0.43
6	C.Fr.U	0.56	-7.85	-1.37	-9.22	-0.56	4.42	3.75	0.29	0.88	1.92	0.51	1.41
7	C.Fr.F	0.48	-6.89	-4.24	-11.13	-1.16	4.33	4.22	0.20	0.75	2.55	0.61	0.78
8	A.En.U	0.48	-1.96	-6.98	-8.95	-1.71	4.28	3.49	0.34	0.01	2.52	0.72	0.81
9	P.En.F	0.43	-1.73	-8.11	-9.84	-1.69	4.15	3.79	0.28	0.11	2.80	0.74	0.53
10	P.Fr.F	0.42	-5.05	-5.18	-10.24	-1.23	4.16	3.14	0.40	1.33	1.93	0.61	1.40
11	P.En.F	0.38	-3.23	-7.00	-10.23	-1.66	4.26	3.53	0.33	0.31	2.69	0.76	0.64
12	C.En.F	0.35	-6.91	-4.76	-11.67	-1.43	4.39	2.69	0.49	1.52	1.95	0.73	1.38
13	C.En.F	0.31	-2.44	-7.20	-9.64	-1.45	3.85	4.09	0.22	0.14	2.83	0.69	0.50
14	P.En.U	0.29	-1.73	-7.69	-9.41	-1.71	3.84	3.60	0.32	0.14	2.78	0.77	0.55
15	P.Fr.F	0.30	-4.18	-5.60	-9.78	-1.32	4.07	3.55	0.32	0.50	2.33	0.66	1.00

Table S10. Calculated thermodynamic data for each of the 23 hydration sites in *apo* COI1 receptor identified by clustering the active-site solvent density distribution. ‘Neat’ represents the pure TIP3P water.

index	type	f_o	E_{sw}	E_{ww}	E_{tot}	E_{ww}^{nbr}	$-TS^e$	N_{nbr}	f_{enc}	N_{sw}^{HB}	N_{ww}^{HB}	f_{ww}^{HB}	$N_{ww,lost}^{HB}$
neat	-	-	0.00	-9.53	-9.53	-1.36	0.00	5.26	0.00	0.00	3.33	0.63	0.00
0	C.Fr.F	0.97	-11.42	0.32	-11.09	-0.21	6.06	1.35	0.74	1.89	0.68	0.51	2.65
1	P.En.F	0.92	-8.36	-2.45	-10.80	-1.71	5.70	1.57	0.70	1.78	1.16	0.74	2.17
2	C.En.F	0.79	-4.61	-4.94	-9.54	-2.04	5.34	2.37	0.55	0.79	2.09	0.88	1.24
3	C.Fr.F	0.76	-6.54	-3.08	-9.63	-1.13	4.91	2.54	0.52	1.86	1.50	0.59	1.83
4	C.En.U	0.72	-4.85	-4.32	-9.17	-2.04	5.07	1.21	0.77	1.81	0.94	0.78	2.39
5	C.Fr.U	0.7	-6.61	-2.50	-9.11	-1.26	4.61	2.13	0.60	0.88	1.40	0.66	1.93
6	C.Fr.F	0.61	-6.96	-4.75	-11.71	-1.27	4.86	3.50	0.33	1.01	2.35	0.67	0.98
7	C.En.F	0.62	-6.28	-4.69	-10.97	-1.51	4.86	2.21	0.58	1.45	1.61	0.73	1.72
8	C.Fr.F	0.59	-8.44	-3.21	-11.65	-1.01	4.80	3.40	0.35	1.45	2.01	0.59	1.32
9	P.En.F	0.55	-7.86	-2.85	-10.71	-1.59	4.88	2.45	0.54	1.44	1.80	0.74	1.53
10	C.Fr.F	0.55	-9.58	-1.60	-11.18	-0.77	4.59	3.74	0.29	0.33	1.97	0.53	1.36
11	C.Fr.F	0.53	-8.14	-3.26	-11.39	-1.01	4.63	3.86	0.27	1.21	2.26	0.59	1.07
12	A.En.U	0.47	-2.47	-6.59	-9.06	-2.08	4.56	3.17	0.40	0.02	2.64	0.83	0.69
13	P.En.U	0.42	-2.68	-6.75	-9.44	-1.59	3.98	3.79	0.28	0.25	2.75	0.73	0.58
14	A.En.U	0.41	-3.70	-5.79	-9.49	-1.67	4.62	3.34	0.37	0.00	2.59	0.78	0.74
15	C.En.F	0.39	-6.24	-3.96	-10.20	-1.73	4.24	2.28	0.57	0.92	1.82	0.80	1.51
16	A.En.F	0.36	-3.90	-7.21	-11.11	-1.61	4.54	4.29	0.18	0.02	3.34	0.78	-0.01
17	C.Fr.F	0.35	-7.89	-3.23	-11.12	-1.17	3.96	2.71	0.48	1.81	1.70	0.63	1.63
18	A.En.U	0.33	-2.42	-6.17	-8.60	-1.79	4.01	2.94	0.44	0.02	2.22	0.76	1.11
19	P.Fr.F	0.32	-6.75	-3.59	-10.34	-0.97	4.15	3.82	0.27	1.14	2.16	0.57	1.17
20	P.En.U	0.32	-4.81	-2.72	-7.53	-1.73	3.89	1.52	0.71	0.79	1.18	0.78	2.15
21	P.En.F	0.3	-5.26	-4.60	-9.86	-1.72	4.58	2.41	0.54	0.50	1.85	0.77	1.48
22	C.Fr.F	0.3	-8.69	-1.70	-10.39	-1.10	4.22	1.73	0.67	1.59	1.14	0.66	2.19

Table S11. Calculated thermodynamic data for each of the 20 hydration sites in *apo* BRI1 receptor identified by clustering the active-site solvent density distribution. 'Neat' represents the pure TIP3P water.

index	type	f_o	E_{sw}	E_{ww}	E_{tot}	E_{ww}^{nbr}	$-TS^e$	N_{nbr}	f_{enc}	N_{sw}^{HB}	N_{ww}^{HB}	f_{ww}^{HB}	$N_{ww,lost}^{HB}$
neat	-	-	0.00	-9.53	-9.53	-1.36	0.00	5.26	0.00	0.00	3.33	0.63	0.00
0	C.En.F	0.64	-6.72	-3.33	-10.05	-2.11	5.30	1.77	0.66	1.31	1.49	0.85	1.84
1	P.En.U	0.6	-3.77	-5.17	-8.94	-1.56	4.65	2.38	0.55	0.56	1.73	0.73	1.60
2	C.En.U	0.55	-4.58	-4.36	-8.93	-2.02	4.88	1.94	0.63	0.95	1.64	0.84	1.69
3	P.Fr.U	0.46	-3.77	-5.63	-9.40	-1.30	4.22	3.17	0.40	1.12	2.05	0.65	1.28
4	P.En.U	0.43	-3.39	-6.01	-9.41	-1.44	3.95	3.05	0.42	0.86	2.08	0.68	1.25
5	P.En.F	0.39	-3.40	-6.25	-9.65	-1.56	4.25	3.35	0.36	0.79	2.39	0.71	0.94
6	A.En.U	0.39	-1.10	-8.07	-9.17	-1.74	3.86	3.66	0.30	0.01	2.82	0.77	0.51
7	P.En.F	0.36	-5.65	-4.82	-10.46	-1.53	4.76	3.04	0.42	0.99	2.27	0.75	1.06
8	P.En.F	0.36	-3.71	-6.36	-10.07	-1.48	4.07	3.15	0.40	1.25	2.05	0.65	1.28
9	P.En.U	0.35	-2.96	-6.01	-8.97	-1.47	3.99	3.54	0.33	0.66	2.32	0.65	1.01
10	P.Fr.U	0.34	-2.95	-6.50	-9.45	-1.32	3.71	3.56	0.32	1.08	2.12	0.59	1.21
11	A.En.U	0.32	-0.95	-7.36	-8.31	-1.73	3.85	3.05	0.42	0.00	2.29	0.75	1.04
12	P.En.U	0.32	-1.48	-8.00	-9.48	-1.44	3.60	4.05	0.23	0.73	2.66	0.66	0.67
13	P.En.U	0.32	-3.34	-5.91	-9.25	-1.39	3.66	3.47	0.34	0.89	2.28	0.66	1.05
14	P.En.F	0.31	-4.45	-5.84	-10.29	-1.79	4.19	2.75	0.48	1.10	2.15	0.78	1.18
15	P.Fr.F	0.31	-3.90	-6.03	-9.93	-1.19	3.76	4.02	0.24	0.87	2.46	0.61	0.87
16	C.Fr.U	0.3	-4.13	-5.22	-9.35	-1.26	3.71	3.28	0.38	0.91	2.00	0.61	1.33
17	A.En.U	0.29	-2.01	-7.36	-9.38	-1.58	3.81	3.76	0.28	0.00	2.66	0.71	0.67
18	P.En.U	0.29	-2.91	-6.29	-9.20	-1.52	3.67	3.17	0.40	0.62	2.25	0.71	1.08
19	A.En.U	0.29	-1.63	-6.02	-7.65	-1.80	3.77	2.64	0.50	0.02	2.03	0.77	1.30

Table S12. Calculated thermodynamic data for each of the 29 hydration sites in *apo* D14 receptor identified by clustering the active-site solvent density distribution. 'Neat' represents the pure TIP3P water.

index	type	f_o	E_{sw}	E_{ww}	E_{tot}	E_{ww}^{nbr}	$-TS^e$	N_{nbr}	f_{enc}	N_{sw}^{HB}	N_{ww}^{HB}	f_{ww}^{HB}	$N_{ww,lost}^{HB}$
neat	-	-	0.00	-9.53	-9.53	-1.36	0.00	5.26	0.00	0.00	3.33	0.63	0.00
0	P.En.F	0.85	-3.60	-5.98	-9.57	-1.82	4.90	2.79	0.47	0.77	2.10	0.75	1.23
1	P.En.F	0.84	-7.04	-2.72	-9.75	-1.85	5.50	1.30	0.75	1.19	1.06	0.81	2.27
2	P.En.F	0.73	-4.91	-4.70	-9.62	-1.79	5.10	2.40	0.54	0.46	1.99	0.83	1.34
3	C.Fr.F	0.67	-7.27	-4.30	-11.58	-0.99	4.96	3.42	0.35	0.96	2.06	0.60	1.27
4	P.En.U	0.69	-3.77	-4.59	-8.35	-2.05	4.81	1.95	0.63	0.55	1.71	0.88	1.62
5	P.En.U	0.65	-5.37	-3.07	-8.44	-2.04	5.14	1.47	0.72	0.74	1.24	0.84	2.09
6	A.En.F	0.64	-2.87	-7.34	-10.20	-1.62	5.46	3.47	0.34	0.00	2.72	0.78	0.61
7	P.En.U	0.53	-1.88	-6.88	-8.75	-1.44	4.30	3.50	0.33	0.32	2.41	0.69	0.92
8	P.En.F	0.52	-3.88	-5.86	-9.75	-1.73	4.79	3.17	0.40	0.51	2.44	0.77	0.89
9	C.Fr.F	0.47	-5.38	-4.51	-9.89	-1.33	4.78	3.11	0.41	0.76	1.89	0.61	1.44
10	P.En.U	0.46	-4.45	-4.82	-9.27	-1.59	4.41	2.36	0.55	0.95	1.79	0.76	1.54
11	A.En.U	0.42	-3.14	-5.68	-8.83	-1.77	4.51	2.76	0.48	0.00	2.11	0.77	1.22
12	A.En.F	0.42	-3.59	-7.32	-10.91	-1.57	4.49	4.29	0.19	0.00	3.35	0.78	-0.02
13	A.En.F	0.42	-1.66	-8.68	-10.34	-1.61	4.66	4.57	0.13	0.00	3.34	0.73	-0.01
14	C.En.F	0.4	-7.44	-3.73	-11.17	-1.51	4.93	2.51	0.52	1.60	1.79	0.72	1.54
15	A.En.U	0.4	-1.19	-7.81	-9.00	-1.69	3.98	3.64	0.31	0.00	2.82	0.77	0.51
16	P.En.F	0.38	-4.08	-5.89	-9.97	-1.52	4.35	2.94	0.44	0.66	2.15	0.73	1.18
17	A.En.U	0.37	-1.53	-7.45	-8.97	-1.72	4.25	3.46	0.34	0.00	2.65	0.77	0.68
18	A.En.U	0.37	-0.78	-8.26	-9.04	-1.56	4.02	3.97	0.24	0.00	2.97	0.75	0.36
19	P.En.U	0.36	-1.59	-7.70	-9.29	-1.65	4.21	3.57	0.32	0.24	2.74	0.77	0.59
20	A.En.U	0.36	-3.39	-6.14	-9.53	-1.61	4.18	3.46	0.34	0.01	2.69	0.78	0.64
21	A.En.U	0.35	-1.09	-8.34	-9.43	-1.62	4.12	4.10	0.22	0.04	3.00	0.73	0.33
22	A.En.F	0.35	-2.97	-6.83	-9.80	-1.72	4.57	3.76	0.28	0.00	2.95	0.78	0.38
23	P.En.U	0.33	-3.26	-4.78	-8.05	-1.44	3.64	2.89	0.45	0.32	1.98	0.68	1.35
24	A.En.F	0.33	-1.58	-8.18	-9.75	-1.64	3.76	4.29	0.18	0.01	3.16	0.74	0.17
25	A.En.F	0.31	-2.27	-7.79	-10.06	-1.83	4.57	3.75	0.29	0.00	3.08	0.82	0.25
26	A.En.F	0.31	-1.69	-7.98	-9.67	-1.44	3.84	4.64	0.12	0.01	3.22	0.69	0.11
27	P.En.U	0.3	-2.80	-6.58	-9.37	-1.50	3.75	3.49	0.34	0.88	2.39	0.68	0.94
28	A.En.U	0.3	-0.80	-8.30	-9.10	-1.53	3.67	4.35	0.17	0.09	2.94	0.68	0.39

Table S13. Calculated thermodynamic data for each of the 22 hydration sites in *apo* GID1 receptor identified by clustering the active-site solvent density distribution. 'Neat' represents the pure TIP3P water.

index	type	f_o	E_{sw}	E_{ww}	E_{tot}	E_{ww}^{nbr}	$-TS^e$	N_{nbr}	f_{enc}	N_{sw}^{HB}	N_{ww}^{HB}	f_{ww}^{HB}	$N_{ww,lost}^{HB}$
neat	-	-	0.00	-9.53	-9.53	-1.36	0.00	5.26	0.00	0.00	3.33	0.63	0.00
0	C.En.F	0.9	-6.23	-4.02	-10.25	-1.37	5.61	2.59	0.51	1.43	1.82	0.70	1.51
1	C.En.F	0.89	-9.57	-2.69	-12.26	-1.41	6.14	2.64	0.50	1.91	1.72	0.65	1.61
2	P.Fr.F	0.85	-9.28	-1.42	-10.69	-0.75	5.42	2.26	0.57	1.03	1.45	0.64	1.88
3	P.En.F	0.74	-6.29	-4.13	-10.42	-1.55	5.92	2.21	0.58	1.10	1.86	0.84	1.47
4	C.En.F	0.73	-6.13	-5.12	-11.24	-1.65	5.30	3.14	0.40	1.18	2.60	0.83	0.73
5	C.En.U	0.69	-2.94	-6.37	-9.31	-1.70	4.80	3.03	0.42	0.71	2.19	0.72	1.14
6	C.Fr.F	0.68	-8.10	-3.39	-11.49	-1.12	5.21	3.13	0.41	1.19	2.06	0.66	1.27
7	C.Fr.F	0.64	-7.37	-2.27	-9.64	-1.13	5.25	2.49	0.53	0.92	1.54	0.62	1.79
8	P.Fr.F	0.63	-5.67	-3.96	-9.63	-1.21	4.63	2.48	0.53	1.05	1.71	0.69	1.62
9	P.Fr.F	0.62	-6.27	-4.06	-10.33	-1.17	4.72	2.92	0.45	1.26	1.83	0.63	1.50
10	C.En.U	0.6	-2.33	-6.16	-8.49	-2.39	4.86	2.08	0.60	0.63	1.92	0.92	1.41
11	P.En.F	0.54	-6.15	-4.15	-10.30	-1.66	4.94	1.98	0.62	1.83	1.40	0.71	1.93
12	P.En.F	0.48	-2.68	-7.05	-9.73	-1.64	4.19	3.69	0.30	0.12	2.83	0.77	0.50
13	P.En.F	0.47	-3.36	-6.65	-10.01	-1.55	4.83	3.36	0.36	0.86	2.68	0.80	0.65
14	P.Fr.U	0.4	-4.41	-5.03	-9.44	-1.20	4.29	3.99	0.24	0.65	2.48	0.62	0.85
15	P.En.F	0.39	-5.16	-4.89	-10.05	-1.38	4.50	3.72	0.29	0.47	2.54	0.68	0.79
16	P.En.U	0.37	-0.91	-6.91	-7.81	-1.87	4.27	2.65	0.50	0.13	1.96	0.74	1.37
17	A.En.U	0.34	-1.63	-7.89	-9.52	-1.96	4.16	3.31	0.37	0.02	2.73	0.83	0.60
18	A.En.U	0.32	-3.34	-5.82	-9.17	-1.51	4.19	3.70	0.30	0.01	2.60	0.70	0.73
19	P.En.U	0.3	-2.91	-5.53	-8.44	-1.53	4.00	2.90	0.45	0.52	2.01	0.69	1.32
20	P.En.F	0.3	-6.38	-3.21	-9.59	-1.66	4.15	1.64	0.69	1.96	1.30	0.80	2.03
21	P.Fr.F	0.3	-8.87	-3.91	-12.78	-1.36	5.25	2.96	0.44	1.61	2.13	0.72	1.20

Table S14. Calculated thermodynamic data for each of the 15 hydration sites in *apo* AHK4 receptor identified by clustering the active-site solvent density distribution. 'Neat' represents the pure TIP3P water.

index	type	f_o	E_{sw}	E_{ww}	E_{tot}	E_{ww}^{nbr}	$-TS^e$	N_{nbr}	f_{enc}	N_{sw}^{HB}	N_{ww}^{HB}	f_{ww}^{HB}	$N_{ww,lost}^{HB}$
neat	-	-	0.00	-9.53	-9.53	-1.36	0.00	5.26	0.00	0.00	3.33	0.63	0.00
0	P.En.F	0.8	-4.80	-6.37	-11.17	-2.04	5.45	2.91	0.45	1.01	2.61	0.90	0.72
1	C.En.U	0.73	-5.79	-3.69	-9.47	-1.56	4.77	2.58	0.51	0.88	2.03	0.78	1.30
2	P.En.F	0.72	-5.00	-5.95	-10.95	-2.14	5.34	2.14	0.59	1.56	1.88	0.88	1.45
3	C.Fr.U	0.58	-7.44	-1.17	-8.60	-0.79	4.94	2.76	0.48	1.24	1.51	0.55	1.82
4	A.En.U	0.57	-2.83	-5.63	-8.46	-1.77	4.42	3.20	0.39	0.00	2.46	0.77	0.87
5	P.En.U	0.56	-1.87	-6.70	-8.58	-2.08	4.53	2.77	0.47	0.12	2.43	0.88	0.90
6	C.Fr.F	0.55	-8.40	-2.42	-10.83	-1.07	4.89	2.56	0.51	1.22	1.56	0.61	1.77
7	P.En.U	0.5	-4.92	-4.42	-9.34	-2.17	4.37	2.10	0.60	0.88	1.77	0.84	1.56
8	P.En.U	0.43	-2.63	-5.36	-8.00	-2.38	4.60	1.84	0.65	0.88	1.70	0.92	1.63
9	C.En.U	0.39	-2.47	-6.02	-8.50	-1.45	4.24	3.27	0.38	0.89	2.21	0.67	1.12
10	C.Fr.F	0.38	-7.64	-2.47	-10.11	-1.16	4.31	2.75	0.48	1.05	1.58	0.57	1.75
11	P.Fr.U	0.33	-8.55	-0.01	-8.56	-0.95	4.10	1.12	0.79	1.52	0.58	0.52	2.75
12	P.En.U	0.31	-2.28	-5.88	-8.16	-1.97	4.06	2.60	0.50	0.36	2.02	0.78	1.31
13	A.Fr.U	0.3	0.16	-5.45	-5.29	-1.10	3.79	4.00	0.24	0.07	2.35	0.59	0.98
14	A.En.U	0.29	-2.62	-4.71	-7.34	-1.99	3.55	2.16	0.59	0.01	1.80	0.83	1.53

Table S15. Calculated thermodynamic data for each of the 4 hydration sites in *apo* NPR4 receptor identified by clustering the active-site solvent density distribution. 'Neat' represents the pure TIP3P water.

index	type	f_o	E_{sw}	E_{ww}	E_{tot}	E_{ww}^{nbr}	$-TS^e$	N_{nbr}	f_{enc}	N_{sw}^{HB}	N_{ww}^{HB}	f_{ww}^{HB}	$N_{ww,lost}^{HB}$
neat	-	-	0.00	-9.53	-9.53	-1.36	0.00	5.26	0.00	0.00	3.33	0.63	0.00
0	C.En.U	0.97	-6.23	-2.14	-8.37	-2.32	4.52	0.90	0.83	1.05	0.62	0.62	2.78
1	C.En.U	0.62	-4.43	-2.14	-6.57	-2.37	3.99	0.93	0.82	0.48	0.78	0.78	2.74
2	P.En.U	0.59	-3.46	-4.27	-7.73	-2.34	4.33	1.86	0.65	0.32	1.39	0.75	2.15
3	P.En.U	0.41	-3.92	-3.80	-7.72	-2.32	4.57	1.54	0.70	0.74	1.22	0.82	2.36

Table S16. Calculated thermodynamic data for each of the 30 hydration sites in *apo* PYL2 receptor identified by clustering the active-site solvent density distribution. 'Neat' represents the pure TIP3P water.

index	type	f_o	E_{sw}	E_{ww}	E_{tot}	E_{ww}^{nbr}	$-TS^e$	N_{nbr}	f_{enc}	N_{sw}^{HB}	N_{ww}^{HB}	f_{ww}^{HB}	$N_{ww,lost}^{HB}$
neat	-	-	0.00	-9.53	-9.53	-1.36	0.00	5.26	0.00	0.00	3.33	0.63	0.00
0	C.Fr.F	0.94	-14.42	1.76	-12.66	-0.29	6.25	2.14	0.59	1.96	1.31	0.61	2.02
1	C.Fr.F	0.88	-13.61	1.57	-12.05	-0.13	6.20	1.26	0.76	2.85	0.50	0.40	2.83
2	C.En.U	0.87	-2.98	-6.17	-9.15	-1.55	4.73	3.00	0.43	0.89	2.29	0.76	1.04
3	C.Fr.F	0.86	-12.32	0.74	-11.58	-0.35	5.67	2.09	0.60	2.05	1.01	0.48	2.32
4	C.Fr.F	0.84	-14.63	3.42	-11.21	0.26	5.72	2.85	0.46	1.77	1.14	0.40	2.19
5	C.Fr.F	0.78	-7.11	-3.17	-10.28	-1.08	5.19	3.35	0.36	1.00	1.98	0.59	1.35
6	C.Fr.F	0.75	-13.62	0.33	-13.30	-0.65	6.49	1.16	0.78	2.91	0.86	0.74	2.47
7	C.En.F	0.72	-4.11	-5.74	-9.84	-1.88	5.15	2.76	0.47	0.96	2.31	0.83	1.02
8	A.En.U	0.69	-1.42	-7.96	-9.38	-1.90	4.25	3.55	0.33	0.06	2.89	0.82	0.44
9	C.Fr.F	0.66	-6.02	-3.66	-9.68	-0.96	4.75	3.42	0.35	0.93	1.83	0.54	1.50
10	C.Fr.F	0.64	-9.01	-0.97	-9.99	-0.85	5.10	1.55	0.71	1.57	0.93	0.60	2.40
11	C.En.U	0.64	-4.27	-4.75	-9.02	-1.41	4.58	2.97	0.43	0.95	2.04	0.69	1.29
12	A.En.U	0.59	-3.36	-5.70	-9.06	-1.63	4.74	3.38	0.36	0.01	2.72	0.80	0.61
13	P.En.U	0.52	-3.29	-6.21	-9.50	-1.53	4.39	3.39	0.35	0.84	2.36	0.69	0.97
14	P.En.F	0.53	-3.28	-6.70	-9.98	-1.64	4.69	3.21	0.39	0.93	2.39	0.74	0.94
15	A.En.U	0.50	-1.86	-7.57	-9.42	-1.66	4.12	3.73	0.29	0.00	2.64	0.71	0.69
16	P.En.U	0.50	-5.87	-3.29	-9.16	-1.58	4.23	1.63	0.69	1.61	1.18	0.72	2.15
17	C.Fr.U	0.49	-10.10	0.79	-9.31	-0.94	5.51	1.06	0.80	1.13	0.89	0.85	2.44
18	A.En.F	0.48	-1.42	-8.15	-9.57	-1.59	3.93	4.36	0.17	0.01	3.20	0.73	0.13
19	A.En.F	0.40	-0.76	-8.77	-9.53	-1.62	3.78	4.45	0.15	0.02	3.22	0.72	0.11
20	C.En.U	0.39	-3.27	-5.20	-8.47	-1.69	4.01	2.65	0.50	0.59	1.96	0.74	1.37
21	C.Fr.F	0.38	-7.36	-2.18	-9.53	-0.97	4.46	3.45	0.34	0.76	2.05	0.60	1.28
22	A.En.F	0.35	-0.94	-8.85	-9.79	-1.63	3.66	4.40	0.16	0.01	3.24	0.74	0.09
23	P.En.U	0.36	-4.94	-4.12	-9.06	-2.28	5.12	1.14	0.78	1.64	1.00	0.87	2.33
24	P.En.F	0.34	-2.08	-7.86	-9.94	-1.67	3.77	4.01	0.24	0.30	3.01	0.75	0.32
25	A.En.U	0.33	-1.08	-8.39	-9.46	-1.74	3.96	4.08	0.23	0.00	3.05	0.75	0.28
26	P.En.U	0.32	-2.46	-6.77	-9.24	-1.51	3.76	3.35	0.36	0.38	2.31	0.69	1.02
27	P.En.F	0.30	-2.58	-7.25	-9.82	-1.43	3.36	3.82	0.27	0.77	2.49	0.65	0.84
28	P.En.F	0.30	-4.27	-5.79	-10.06	-2.21	5.09	2.02	0.62	1.26	1.84	0.91	1.49
29	A.En.U	0.28	-1.09	-8.22	-9.31	-1.63	3.41	4.09	0.22	0.02	3.13	0.76	0.20

4 Markov State Models Validation

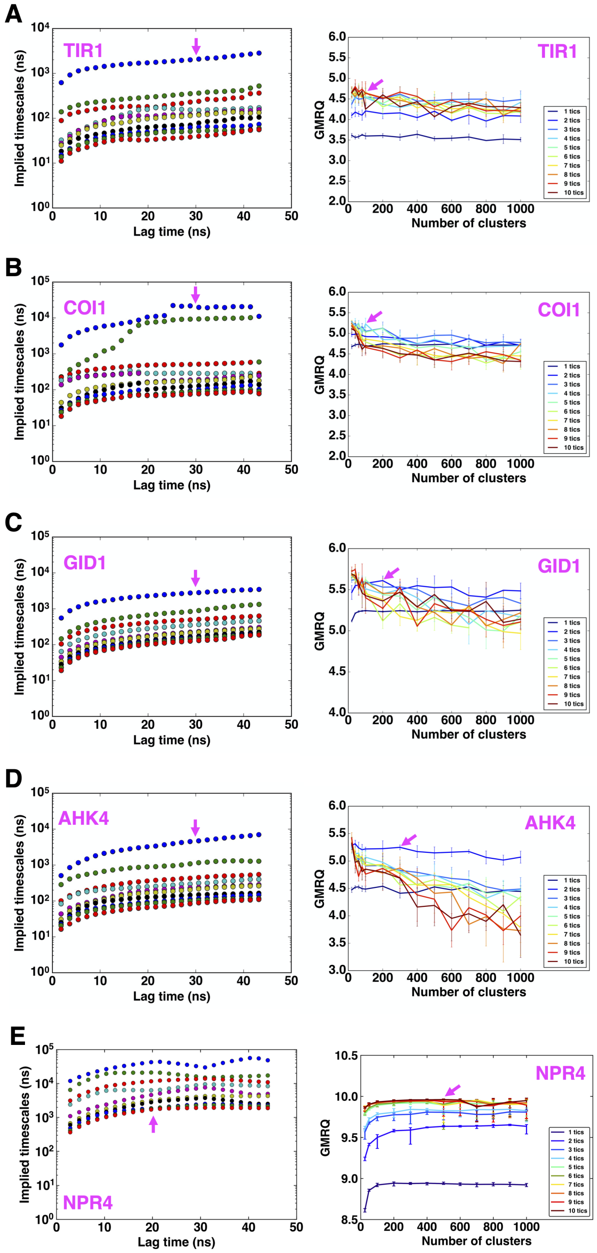

Fig. S1. Cross validation for Markov state models (MSMs) hyperparameter selection. Left panels: the slowest 10 implied timescales of MSMs constructed with increasing lag times converge after 20–30 ns for (A) IAA binding to TIR1, (B) JA-Ile binding to COI1, (C) GA3 binding to GID1, (D) trans-zeatin binding to AHK4, and (E) SA binding to NPR4. Right panels: the GMRQ scores for MSMs constructed with different number of clusters and number of tICs for each system (A–E). The scores were used to select the final MSMs used to describe the binding processes.

5 Binding Pathways

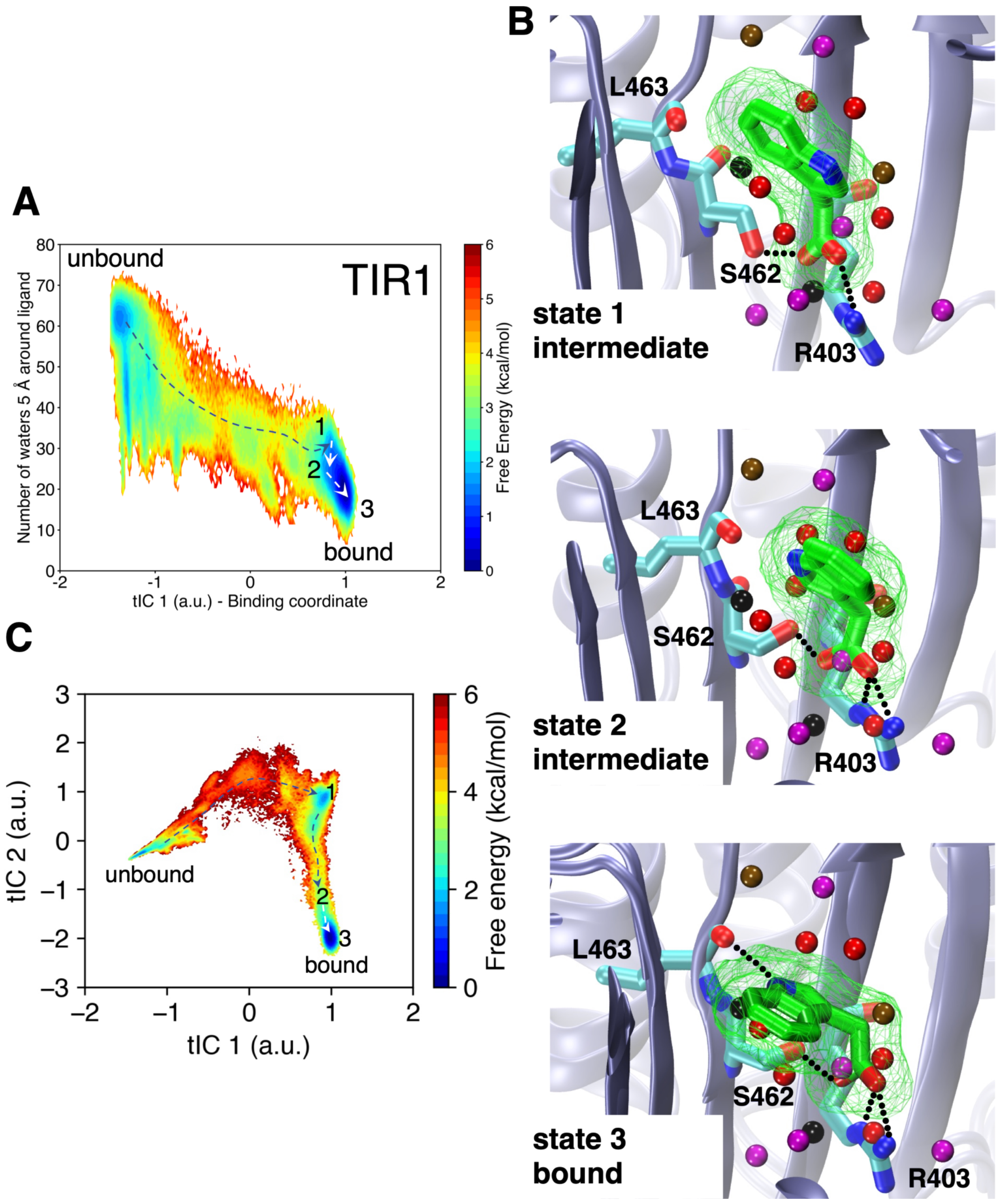

Fig. S2. (A) Free energy landscape of IAA binding to TIR1. The horizontal coordinate is the slowest process recovered from time-lagged independent component analysis (tICA) and is associated with ligand binding, while the vertical coordinate represents the ligand solvation. (Reused panel from Figure 2A.) (B) The snapshots of the binding pathway for the binding of IAA to TIR1. The *apo* hydration sites are shown to indicate the exclusion of water molecules along the IAA binding process. (C) Free energy landscape of IAA binding to TIR1 in terms of the first two tICs.

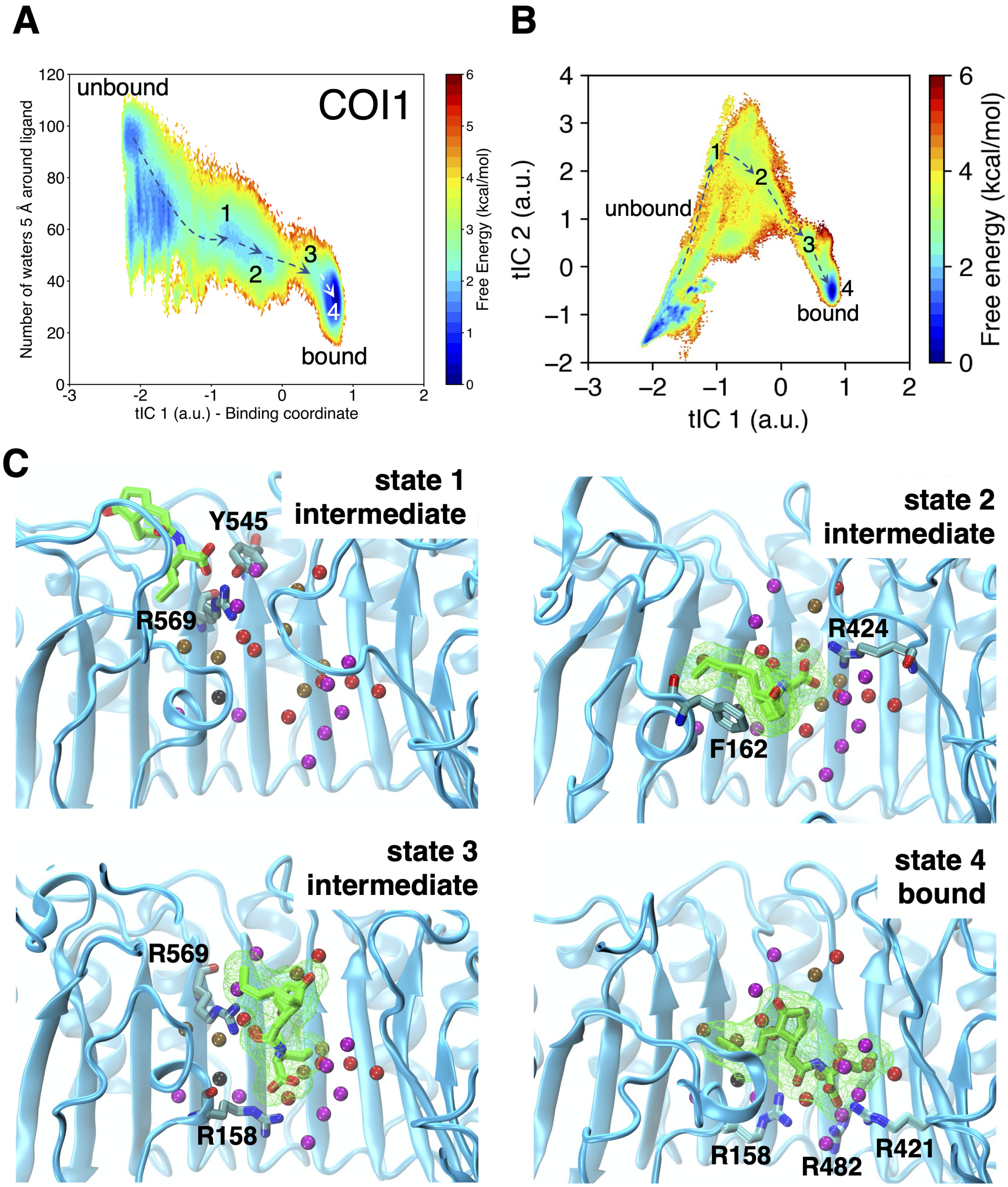

Fig. S3. (A) Free energy landscape of JA-Ile binding to COI1. The horizontal coordinate is the slowest process recovered from time-lagged independent component analysis (tICA) and is associated with ligand binding, while the vertical coordinate represents the ligand solvation. (Reused panel from Figure 2B.) (B) Free energy landscape of JA-Ile binding to COI1 in terms of the first two tICs. (C) The snapshots of the binding pathway for the binding of JA-Ile to COI1. The *apo* hydration sites are shown to indicate the exclusion of water molecules along the JA-Ile binding process.

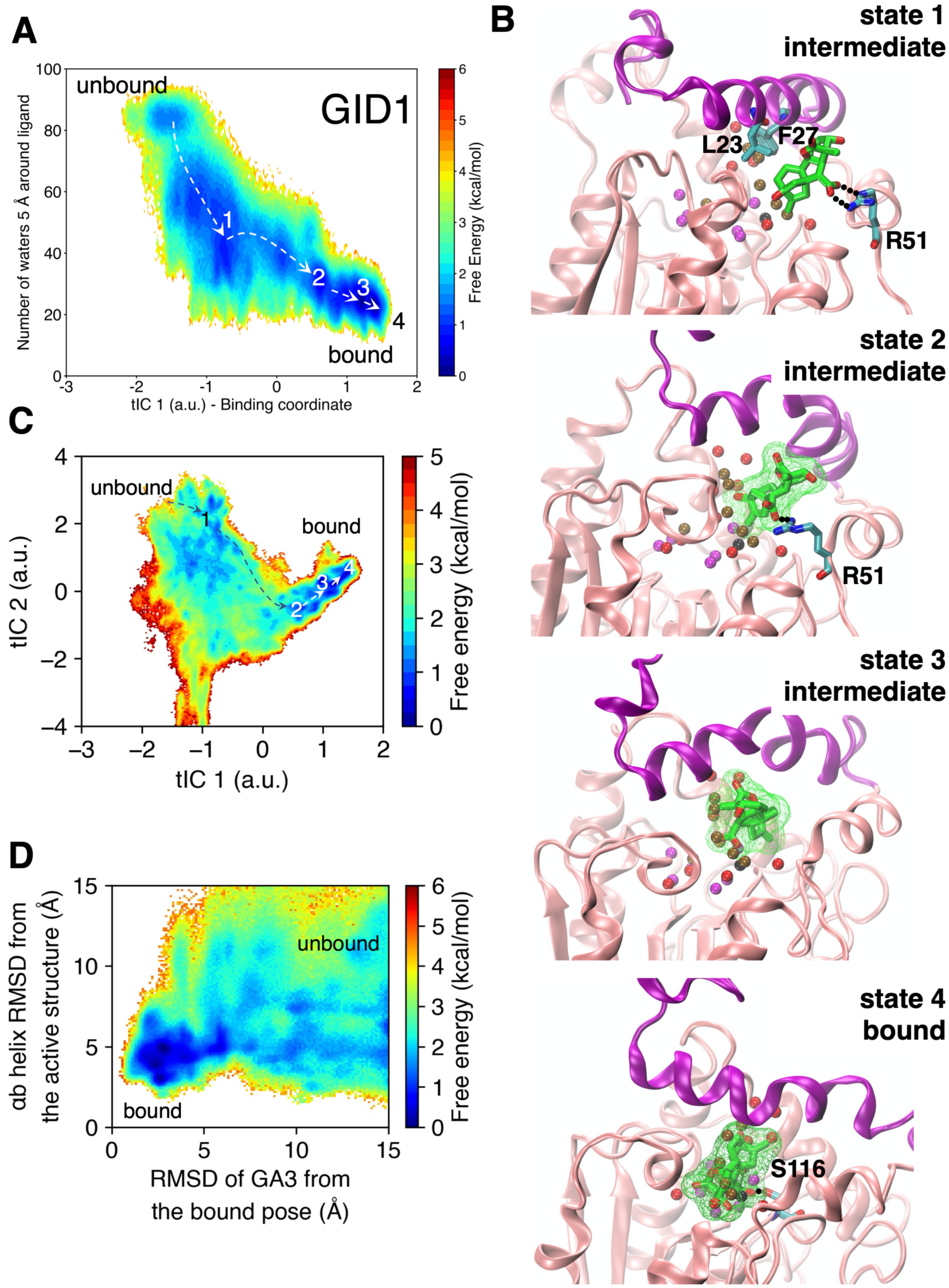

Fig. S4. (A) Free energy landscape of GA3 binding to GID1. The horizontal coordinate is the slowest process recovered from time-lagged independent component analysis (tICA) and is associated with ligand binding, while the vertical coordinate represents the ligand solvation. (Reused panel from Figure 2C.) (B) The snapshots of the binding pathway for the binding of GA3 to GID1. The *apo* hydration sites are shown to indicate the exclusion of water molecules along the GA3 binding process. (C) Free energy landscape of GA3 binding to GID1 in terms of the first two tICs. (D) The binding of GA3 stabilizes the closure of αb helix in the N-terminal of GID1.

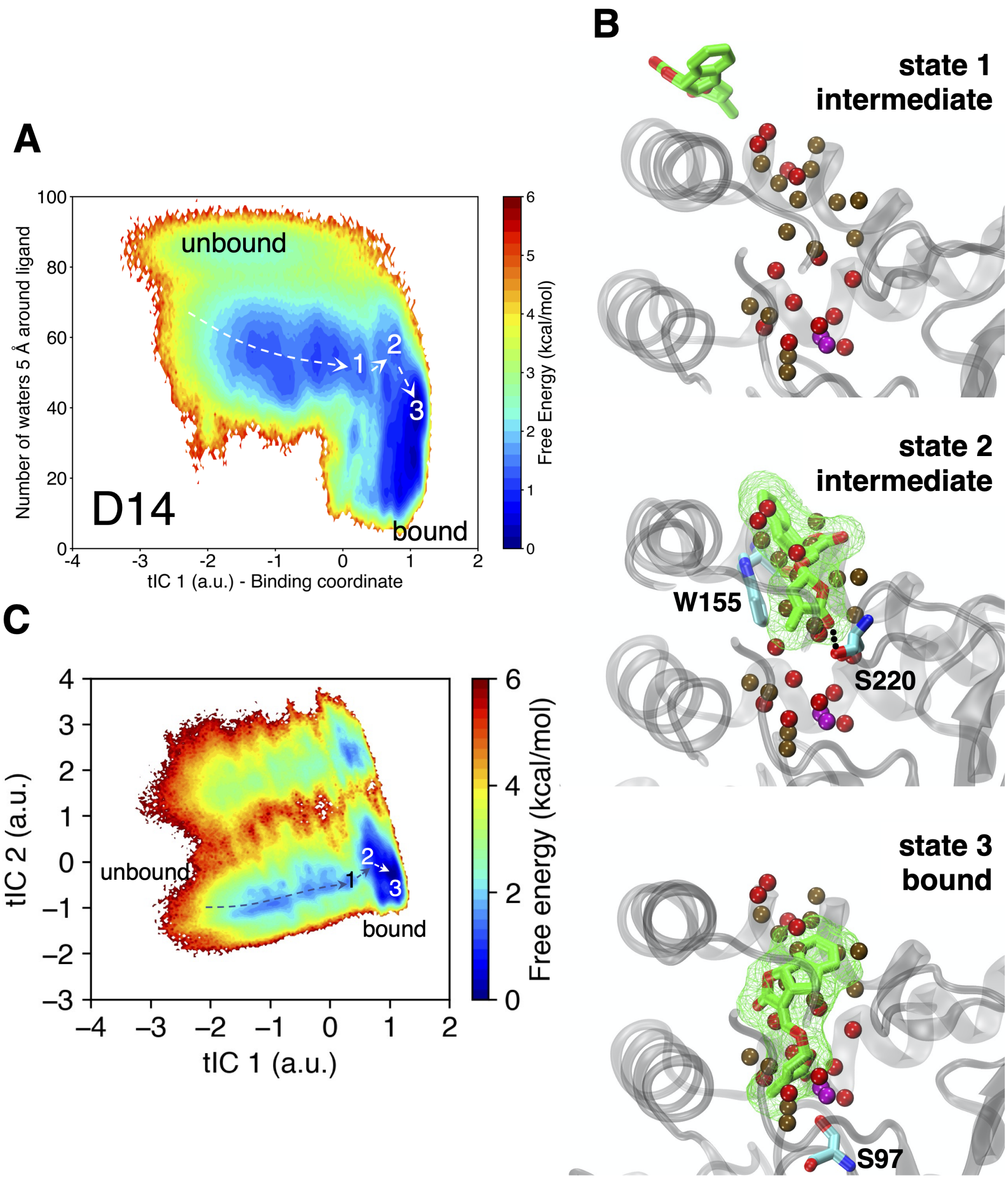

Fig. S5. (A) Free energy landscape of GR24 binding to D14. The horizontal coordinate is the slowest process recovered from time-lagged independent component analysis (tICA) and is associated with ligand binding, while the vertical coordinate represents the ligand solvation. (Reused panel from Figure 2D.) (B) The snapshots of the binding pathway for the binding of GR24 to D14. The *apo* hydration sites are shown to indicate the exclusion of water molecules along the GR24 binding process. (C) Free energy landscape of GR24 binding to D14 in terms of the first two tICs.

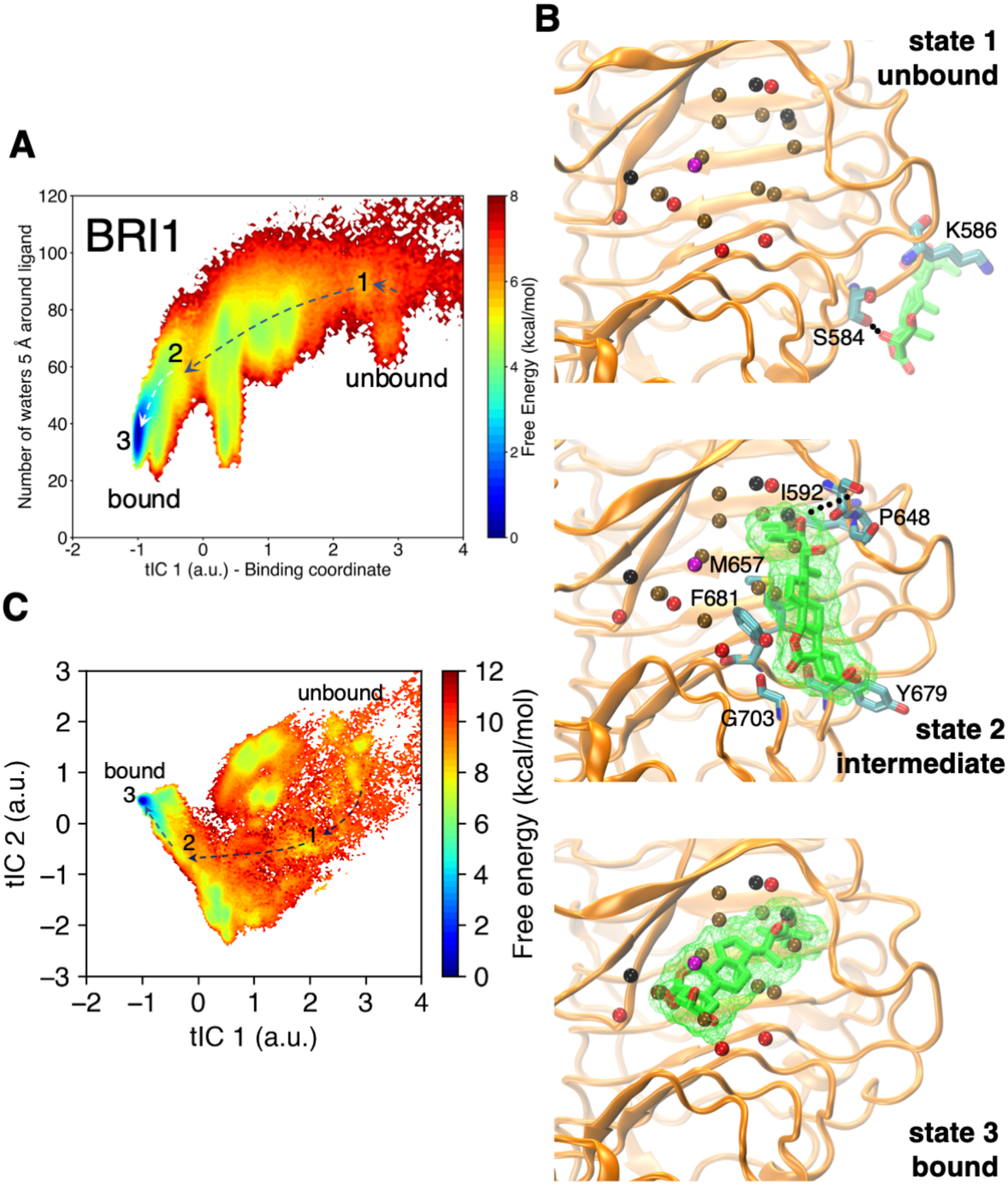

Fig. S6. (A) Free energy landscape of BLD binding to BRI1. The horizontal coordinate is the slowest process recovered from time-lagged independent component analysis (tICA) and is associated with ligand binding, while the vertical coordinate represents the ligand solvation. (Reused panel from Figure 2E.) (B) The snapshots of the binding pathway for the binding of BLD to BRI1. The *apo* hydration sites are shown to indicate the exclusion of water molecules along the BLD binding process. (C) Free energy landscape of GR24 binding to D14 in terms of the first two tICs.

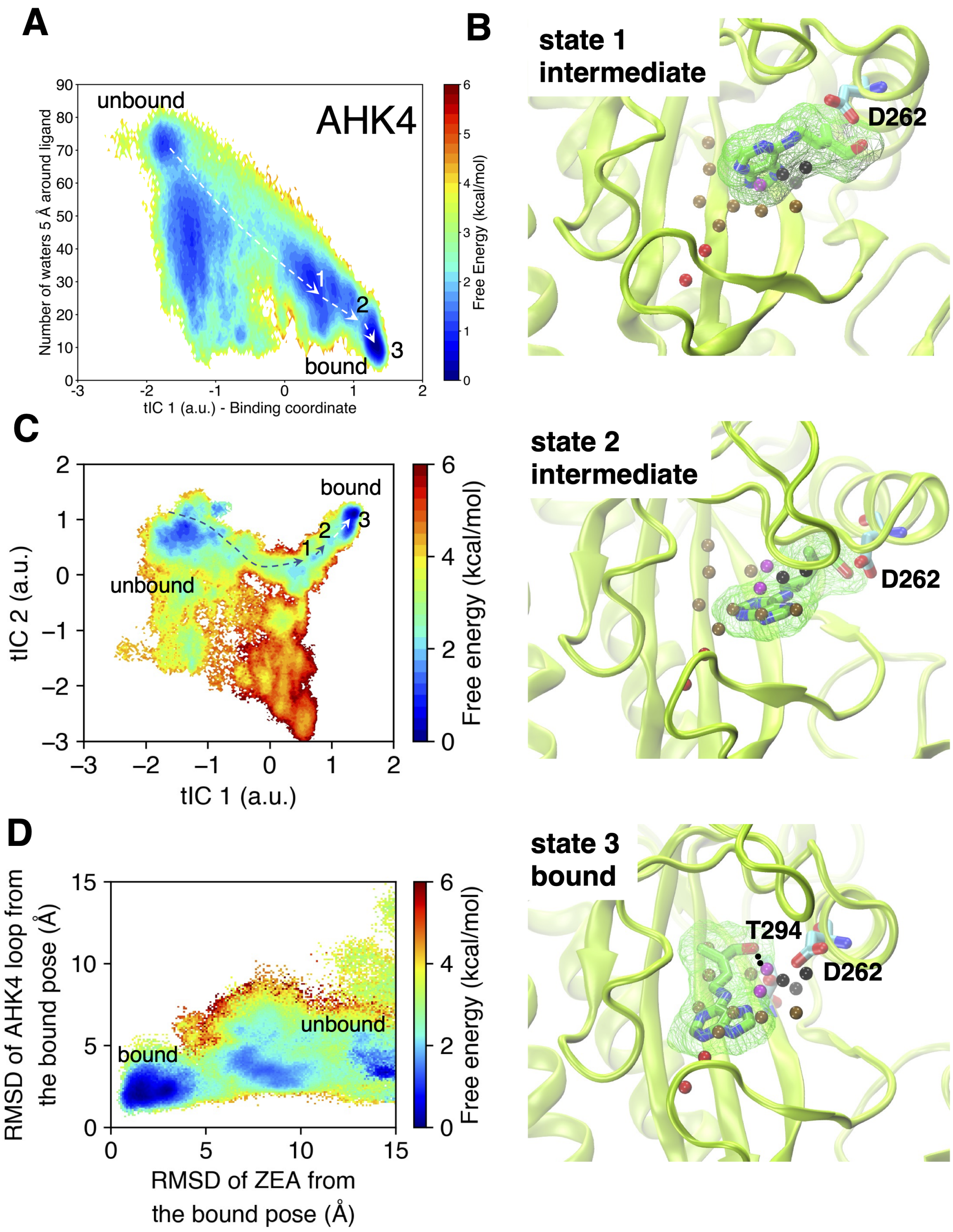

Fig. S7. (A) Free energy landscape of trans-Zeatin binding to AHK4. The horizontal coordinate is the slowest process recovered from time-lagged independent component analysis (tICA) and is associated with ligand binding, while the vertical coordinate represents the ligand solvation. (Reused panel from Figure 2F.) (B) The snapshots of the binding pathway for the binding of trans-Zeatin to AHK4. The *apo* hydration sites are shown to indicate the exclusion of water molecules along the trans-Zeatin binding process. (C) Free energy landscape of trans-Zeatin binding to AHK4 in terms of the first two tICs. (D) The loop in AHK4 that encloses the binding site undergoes conformational changes during the binding of trans-Zeatin. The bound ligand stabilizes the loop after trans-Zeatin is bound to AHK4.

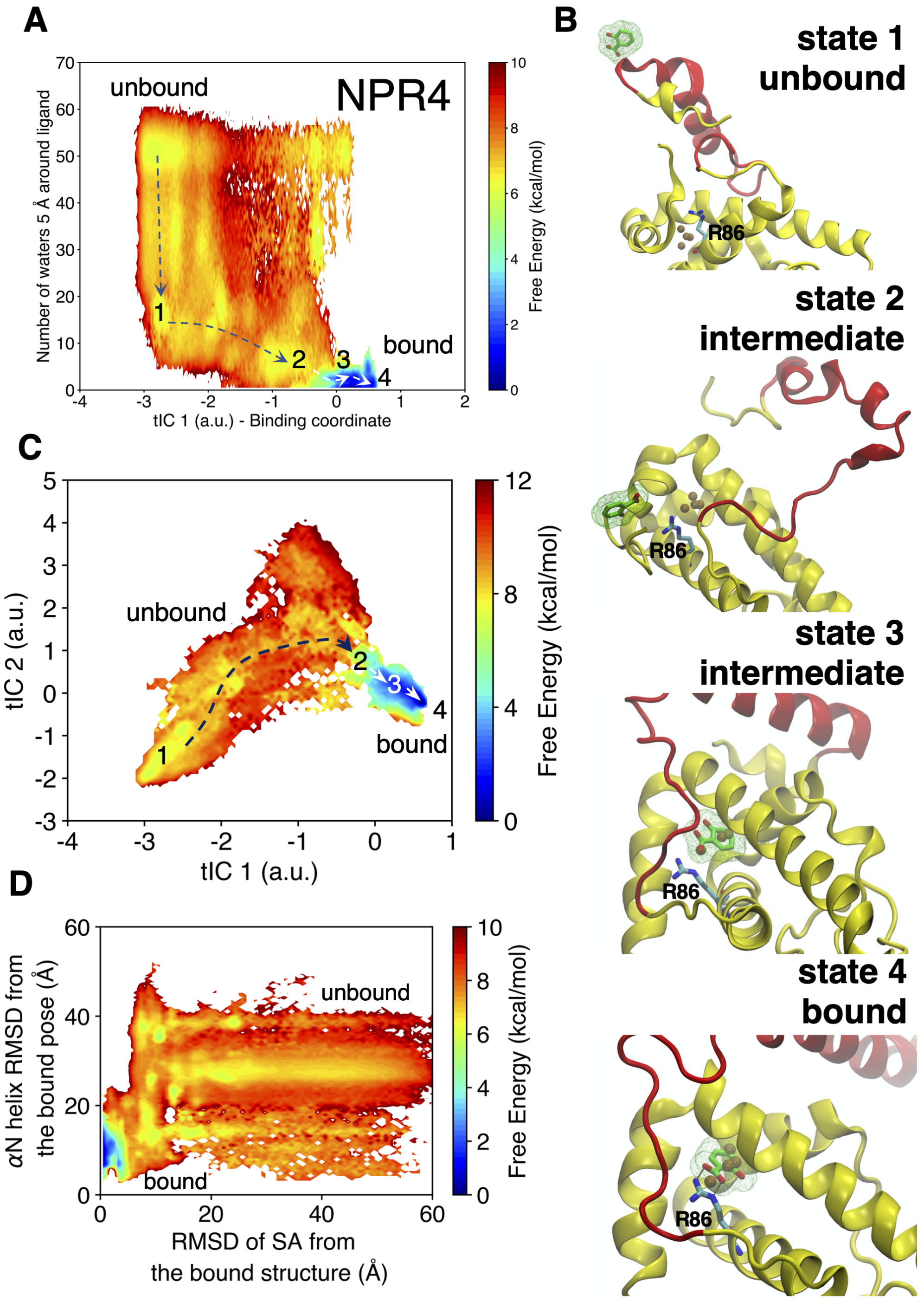

Fig. S8. (A) Free energy landscape of SA binding to the salicylic acid binding core (SBC) in NPR4. The horizontal coordinate is the slowest process recovered from time-lagged independent component analysis (tICA) and is associated with ligand binding, while the vertical coordinate represents the ligand solvation. (Reused panel from Figure 2G.) (B) The snapshots of the binding pathway for the binding of SA to NPR4. The *apo* hydration sites are shown to indicate the exclusion of water molecules along the SA binding process. Unlike other phytohormones, SA displaces all the water molecules from the pocket upon ligand binding. (D) The binding of SA likely stabilizes the closure of the αN helix and loop (shown in red) in the N-terminal of the SBC.

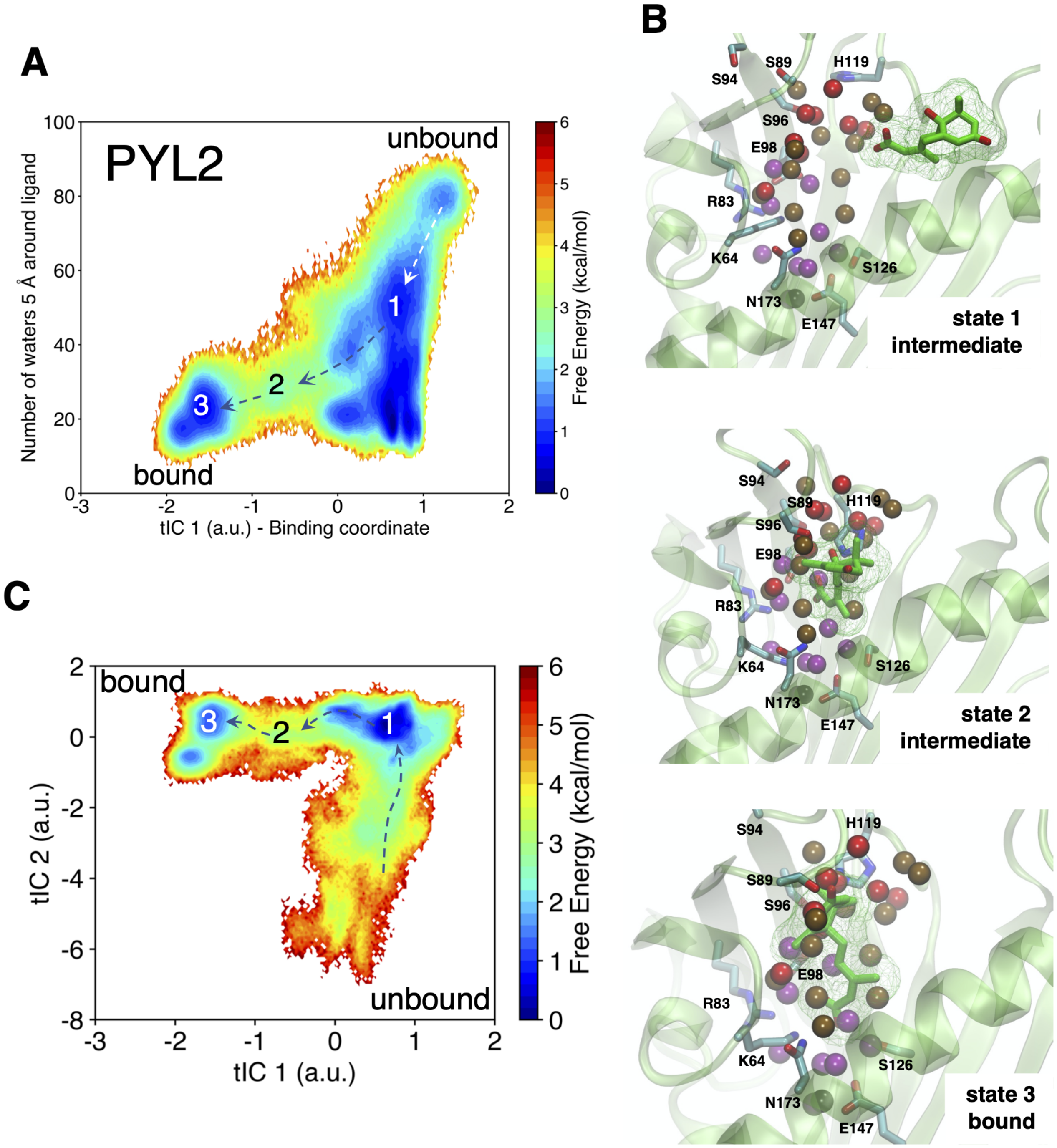

Fig. S9. (A) Free energy landscape of ABA binding to PYL2. The horizontal coordinate is the slowest process recovered from time-lagged independent component analysis (tICA) and is associated with ligand binding, while the vertical coordinate represents the ligand solvation. (Reused panel from Figure 2H.) (B) The snapshots of the binding pathway for the binding of abscisic acid (ABA) to PYL2. The *apo* hydration sites are shown to indicate the exclusion of water molecules along the ABA binding process. (C) Free energy landscape of ABA binding to PYL2 in terms of the first two tICs.

6 Thermodynamic Properties of Hydration Sites

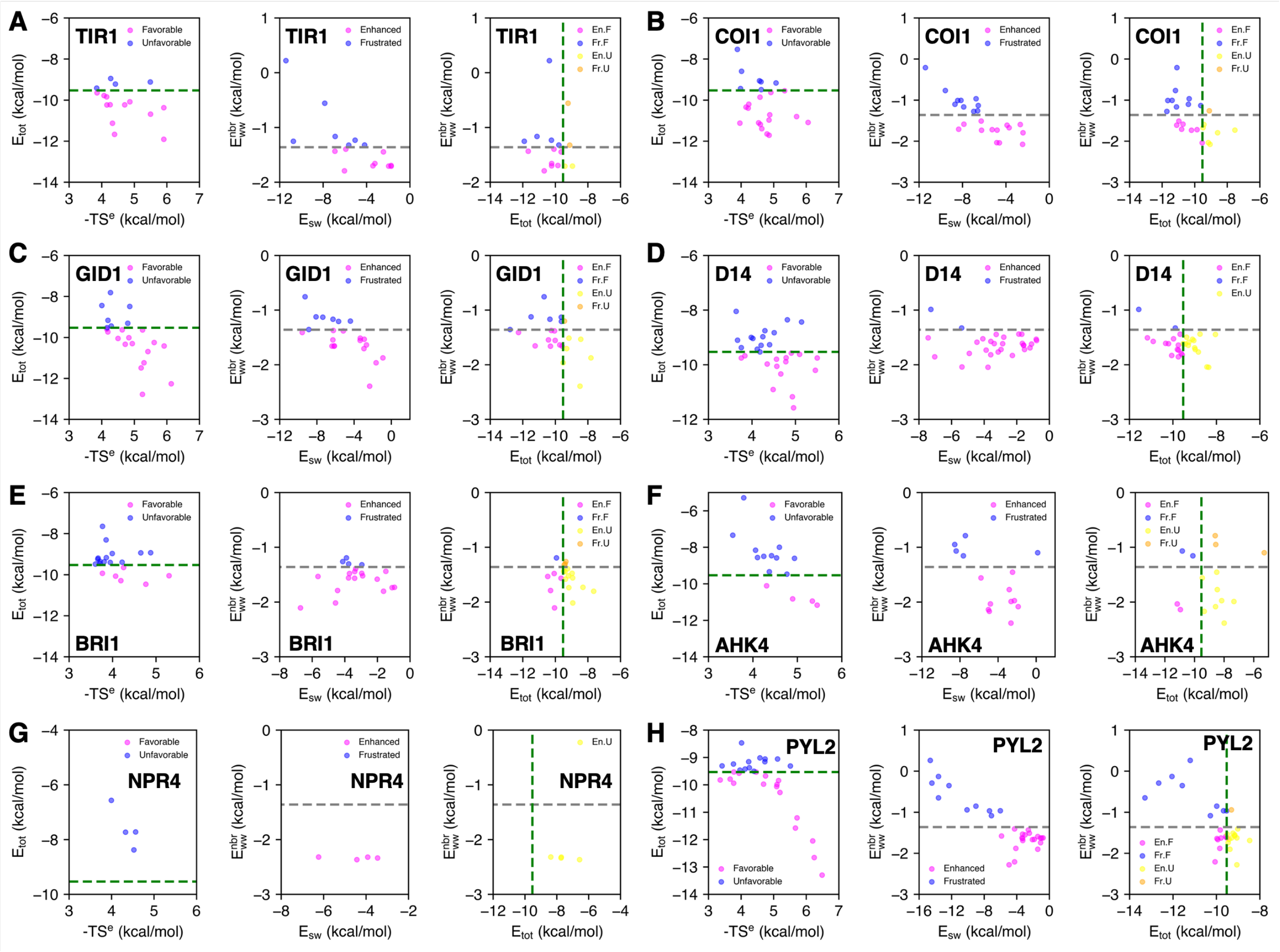

Fig. S10. Thermodynamic properties of *apo* hydration sites in (A) TIR1, (B) COI1, (C) GID1, (D) D14, (E) BRI1, (F) AHK4, (G) NPR4, and (H) PYL2. (Left) E_{tot} and -TS^e of the favorable (magenta) and unfavorable (blue) hydration sites (panels reused from Figure 4), (middle) E_{ww}^{nbr} and E_{sw} of the enhanced (magenta) and frustrated (blue) hydration sites, and (right) E_{ww}^{nbr} and E_{tot} of En.F (magenta), Fr.F (blue), En.U (yellow) and Fr.U (orange) hydration sites.

7 Comparison of Hydration Site Analysis to Crystal Structures

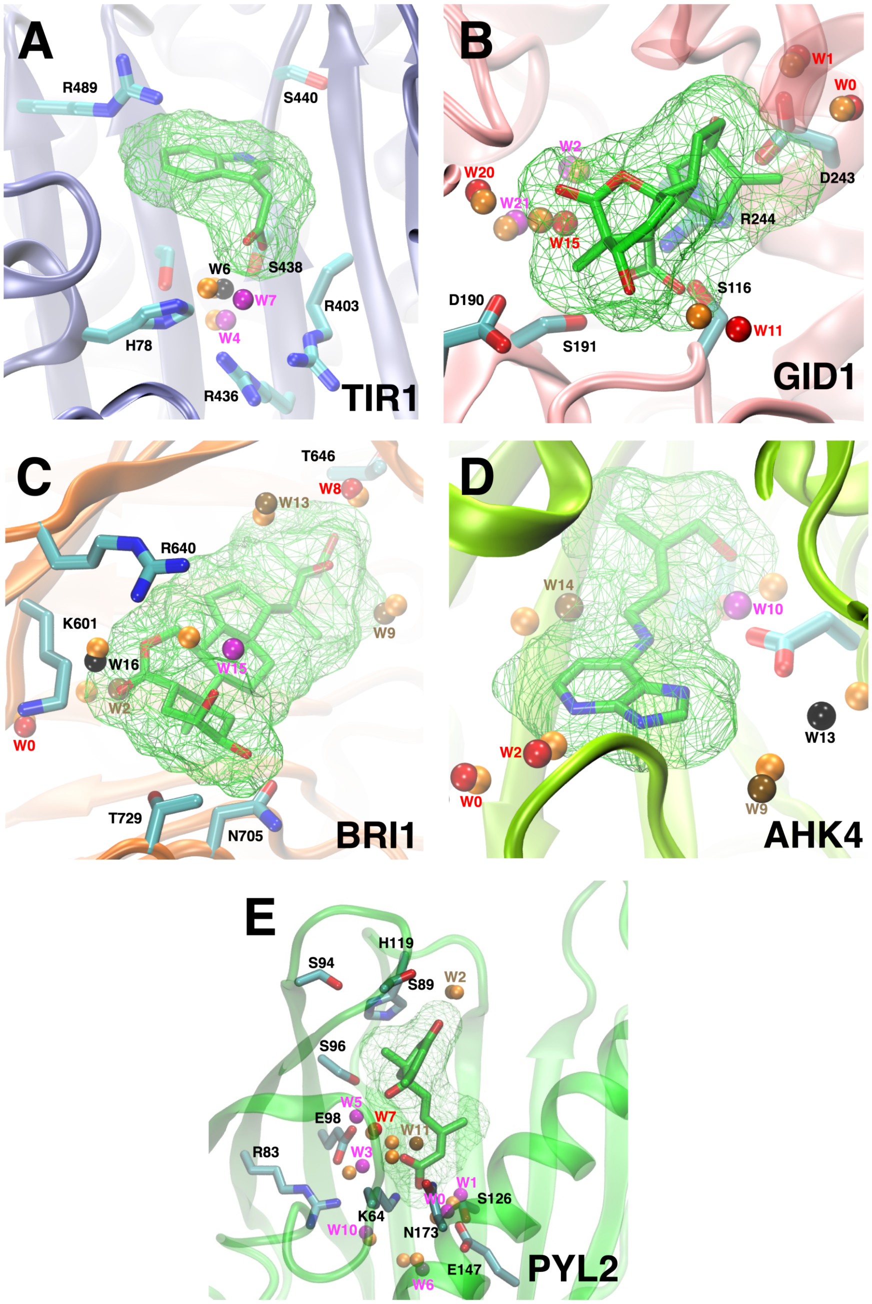

Fig. S11. Overlay of *apo* hydration sites identified from MD simulations (En.F: red, En.U: ochre, Fr.F: magenta, Fr.U: black) and water molecules captured in the crystal structure (orange) of the receptor-hormone bound complexes. (A) TIR1, (B) GID1, (C) BRI1, (D) AHK4, and (E) PYL2.

8 Comparison of *Holo* and *Apo* Hydration Sites

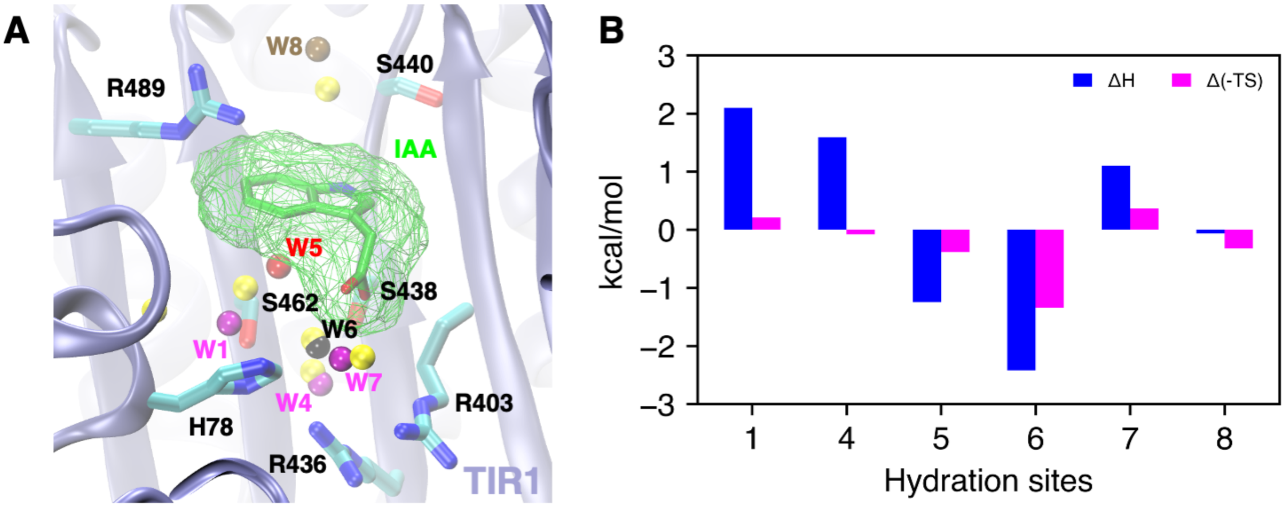

Fig. S12. (A) Overlay of the conserved *apo* hydration sites (labelled) and the corresponding *holo* sites (yellow) in the binding site of TIR1 upon the binding of IAA, and (B) the changes in enthalpy and entropy of conserved hydration sites.

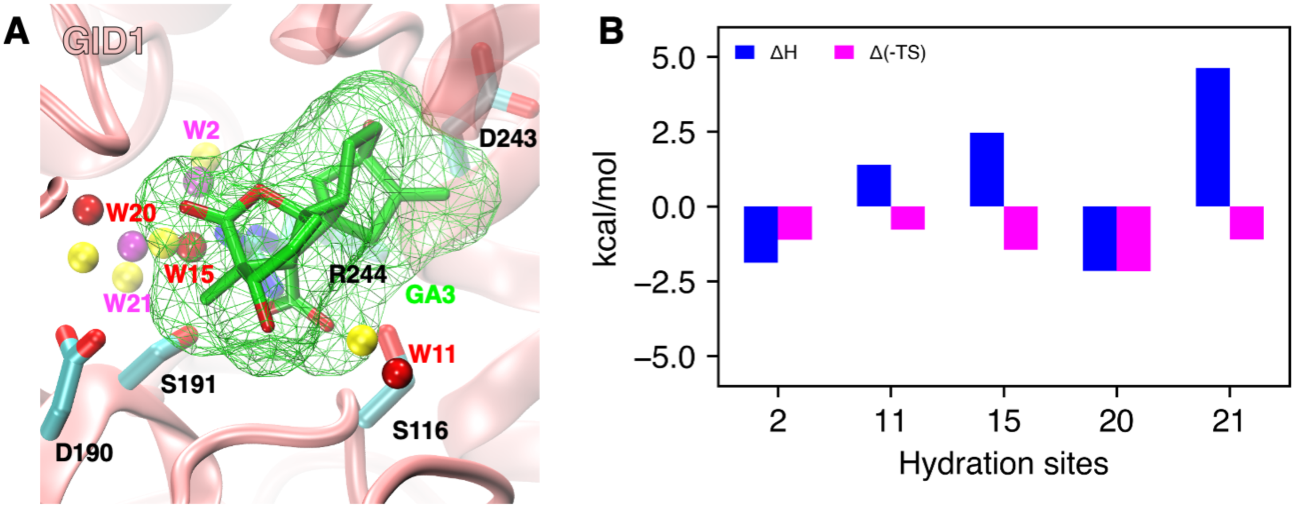

Fig. S13. (A) Overlay of the conserved *apo* hydration sites (labelled) and the corresponding *holo* sites (yellow) in the binding site of GID1 upon the binding of GA3, and (B) the changes in enthalpy and entropy of conserved hydration sites.

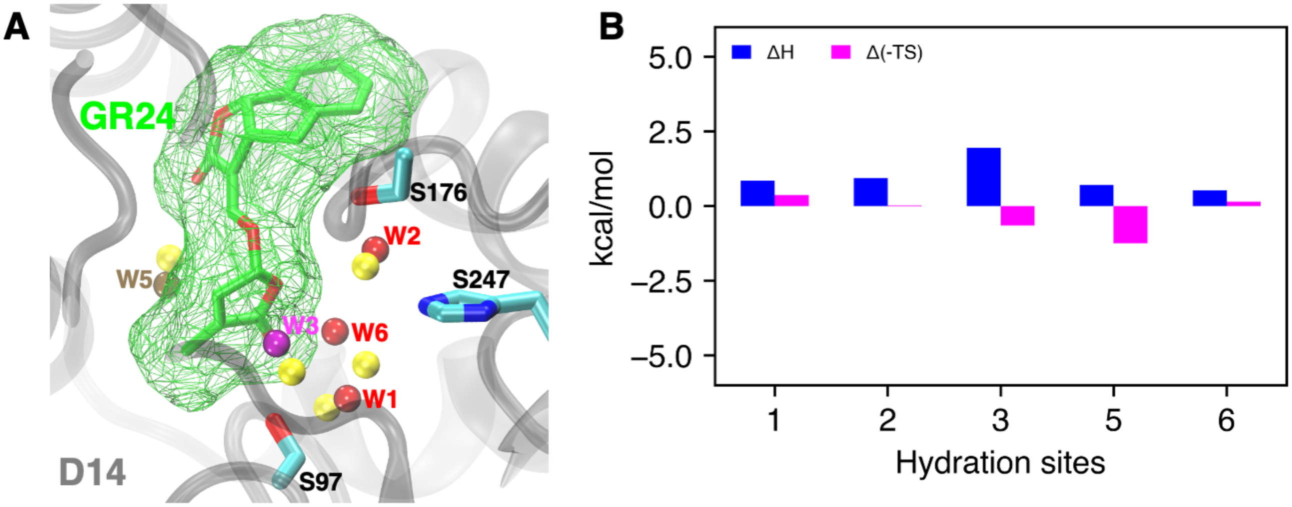

Fig. S14. (A) Overlay of the conserved *apo* hydration sites (labelled) and the corresponding *holo* sites (yellow) in the binding site of D14 upon the binding of GR24, and (B) the changes in enthalpy and entropy of conserved hydration sites.

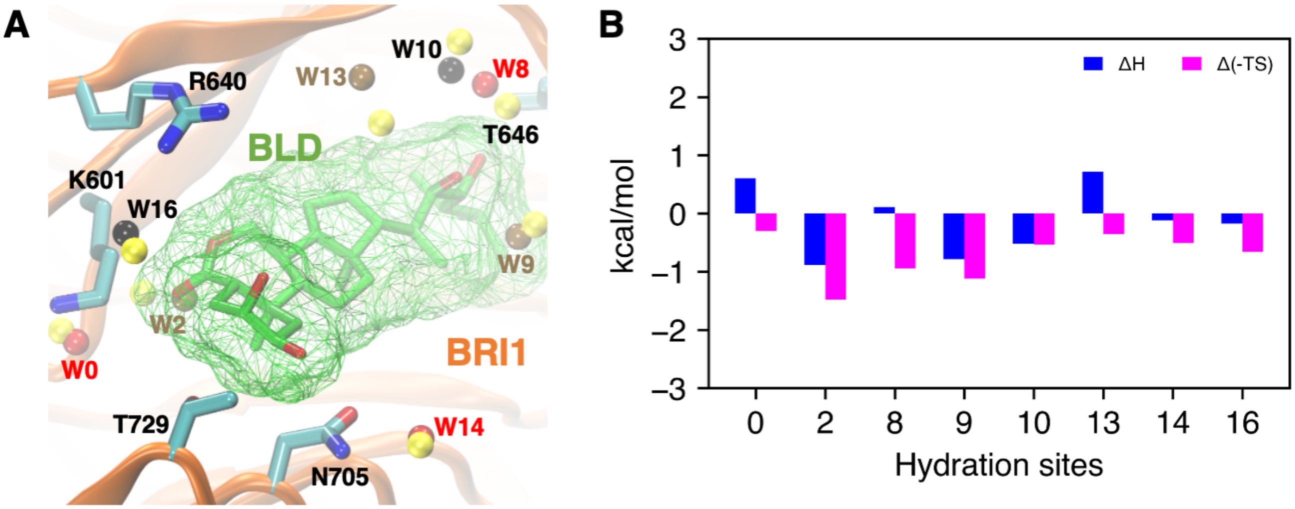

Fig. S15. (A) Overlay of the conserved *apo* hydration sites (labelled) and the corresponding *holo* sites (yellow) in the binding site of BRI1 upon the binding of BLD, and (B) the changes in enthalpy and entropy of conserved hydration sites.

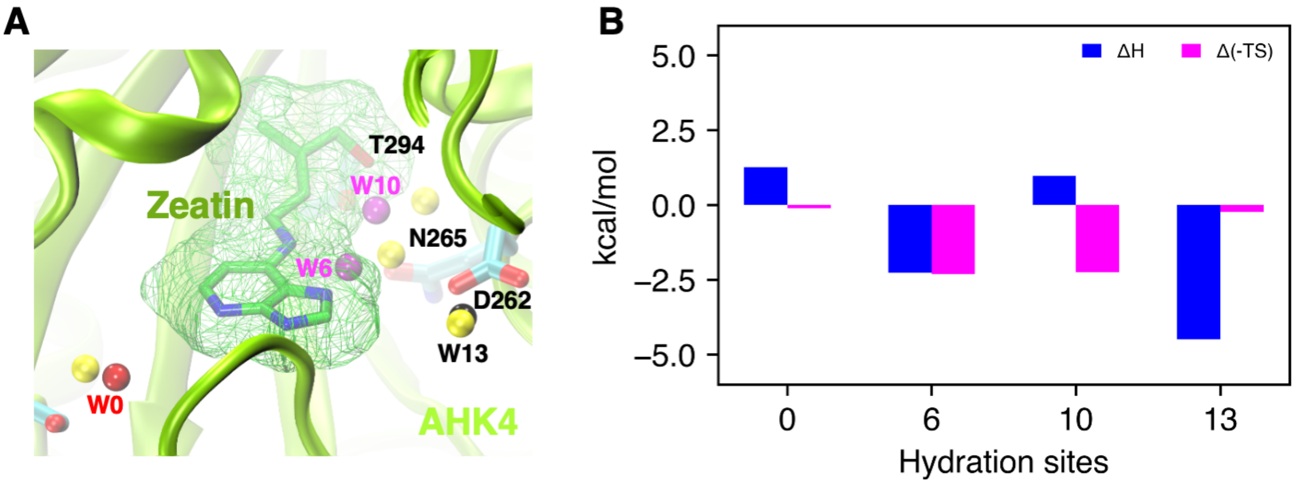

Fig. S16. (A) Overlay of the conserved *apo* hydration sites (labelled) and the corresponding *holo* sites (yellow) in the binding site of AHK4 upon the binding of trans-Zeatin, and (B) the changes in enthalpy and entropy of conserved hydration sites.

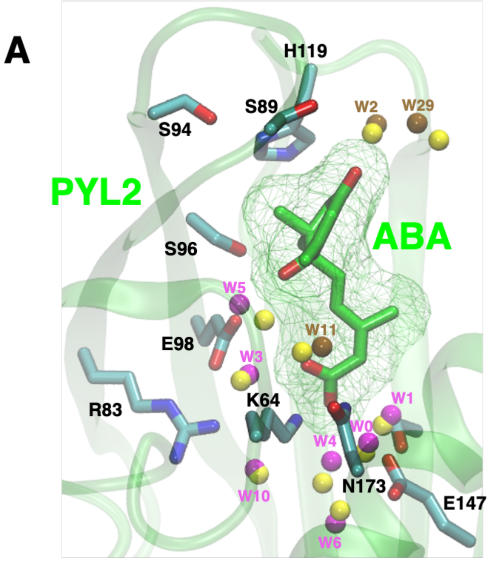

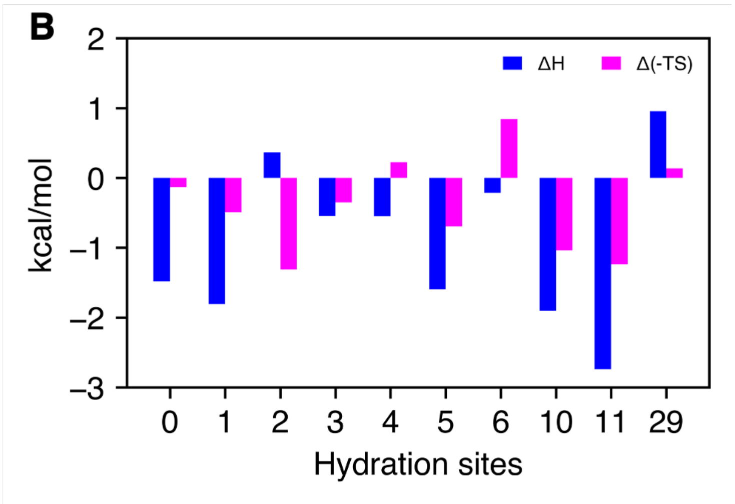

Fig. S17. (A) Overlay of the conserved *apo* hydration sites (labelled) and the corresponding *holo* sites (yellow) in the binding site of PYL2 upon the binding of ABA, and (B) the changes in enthalpy and entropy of conserved hydration sites.

9 Comparison of Abscisic Acid to Pyrabactin in PYL2

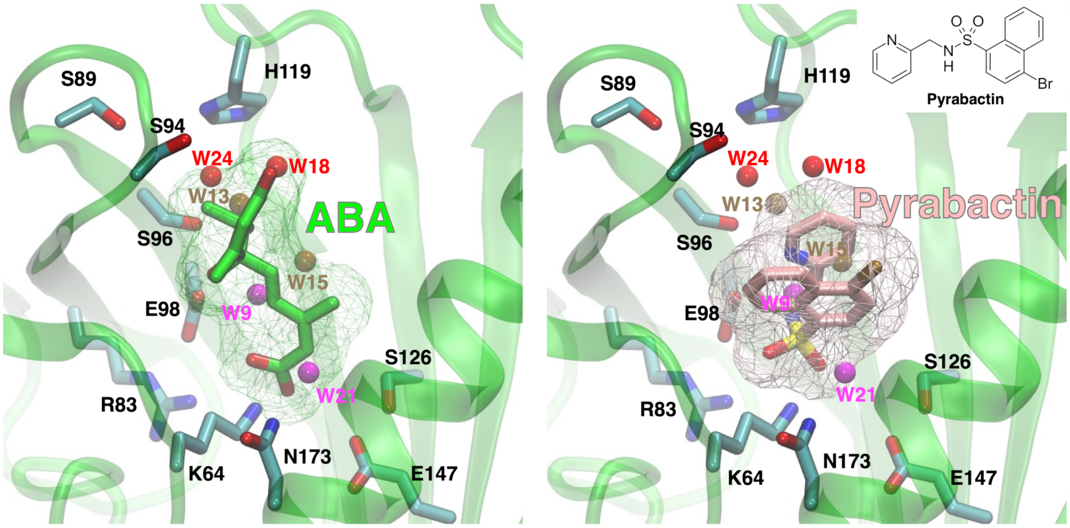

Fig. S18. Hydration site analysis reveals different waters are displaced by ABA and pyrabactin upon binding to PYL2. Left: overlay of ABA and *apo* hydration sites in PYL2. Right: overlay of pyrabactin and *apo* hydration sites in PYL2.

10 Hydrogen Bond Analysis

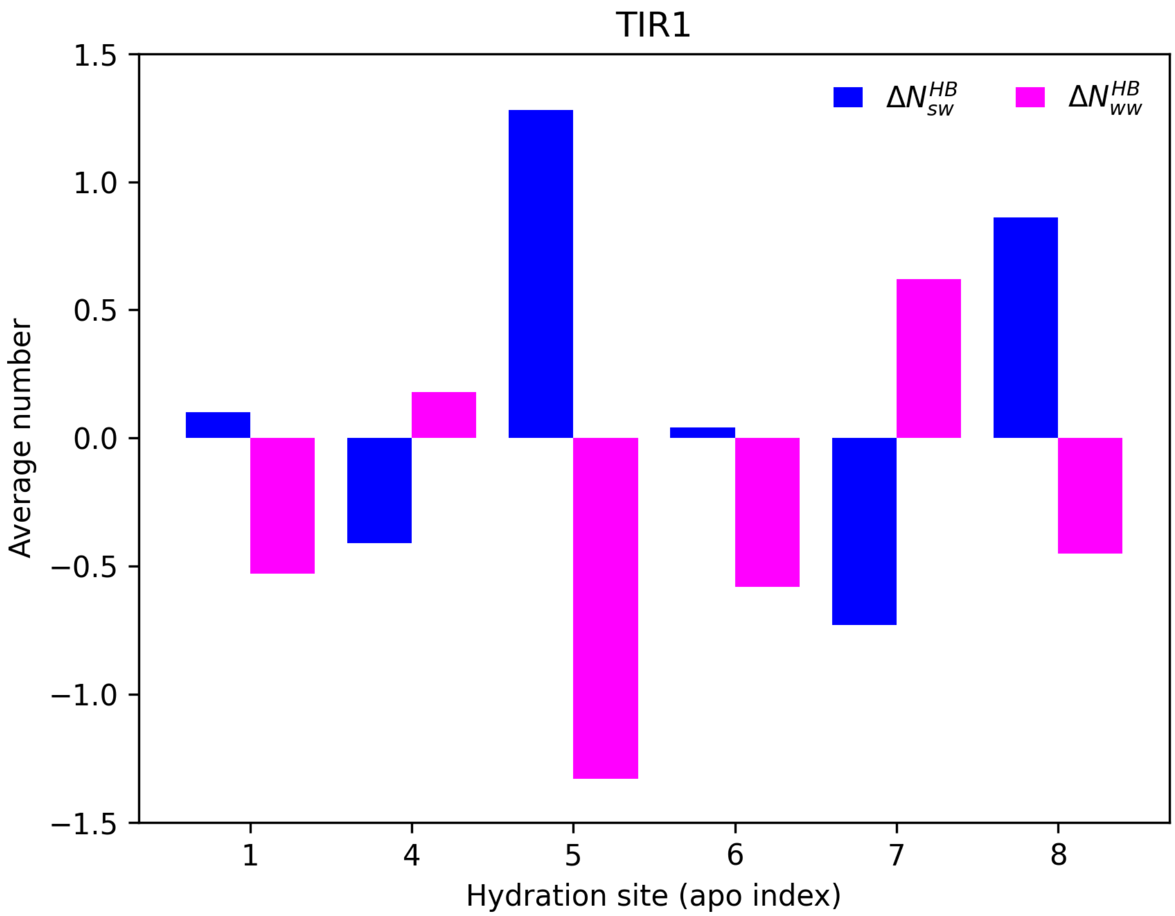

Fig. S19. Difference in hydrogen bonding in conserved *apo* hydration sites and the corresponding *holo* sites in the binding site of TIR1 upon ligand binding. N^{HB}_{sw} is the average number of hydrogen bonds between water and solute molecules. N^{HB}_{ww} is the average number of hydrogen bonds between water molecules. Differences were defined between *holo* and *apo* states: $\Delta N^{HB}_{sw} = N^{HB}_{sw,holo} - N^{HB}_{sw,apo}$ and $\Delta N^{HB}_{ww} = N^{HB}_{ww,holo} - N^{HB}_{ww,apo}$.

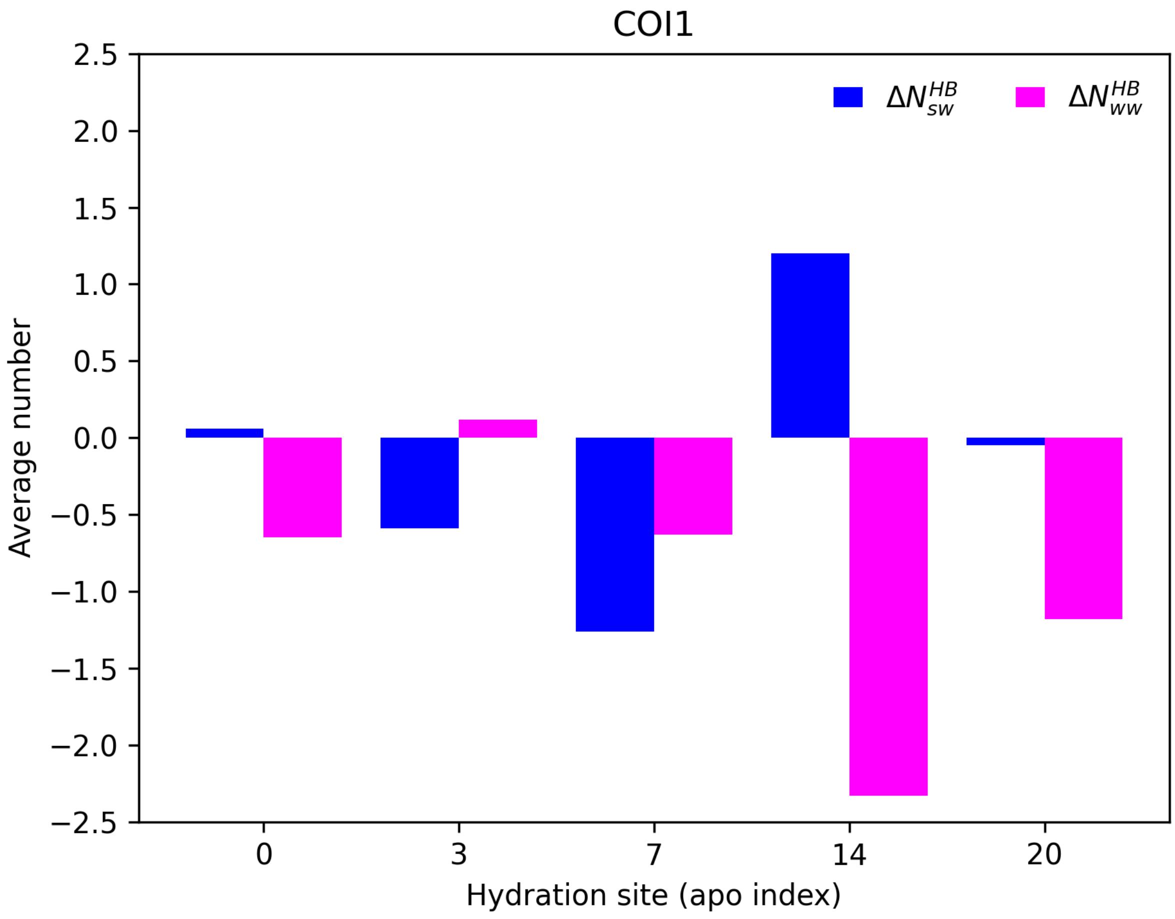

Fig. S20. Difference in hydrogen bonding in conserved *apo* hydration sites and the corresponding *holo* sites in the binding site of COI1 upon ligand binding. N_{sw}^{HB} is the average number of hydrogen bonds between water and solute molecules. N_{ww}^{HB} is the average number of hydrogen bonds between water molecules. Differences were defined between *holo* and *apo* states: $\Delta N_{sw}^{HB} = N_{sw,holo}^{HB} - N_{sw,apo}^{HB}$ and $\Delta N_{ww}^{HB} = N_{ww,holo}^{HB} - N_{ww,apo}^{HB}$.

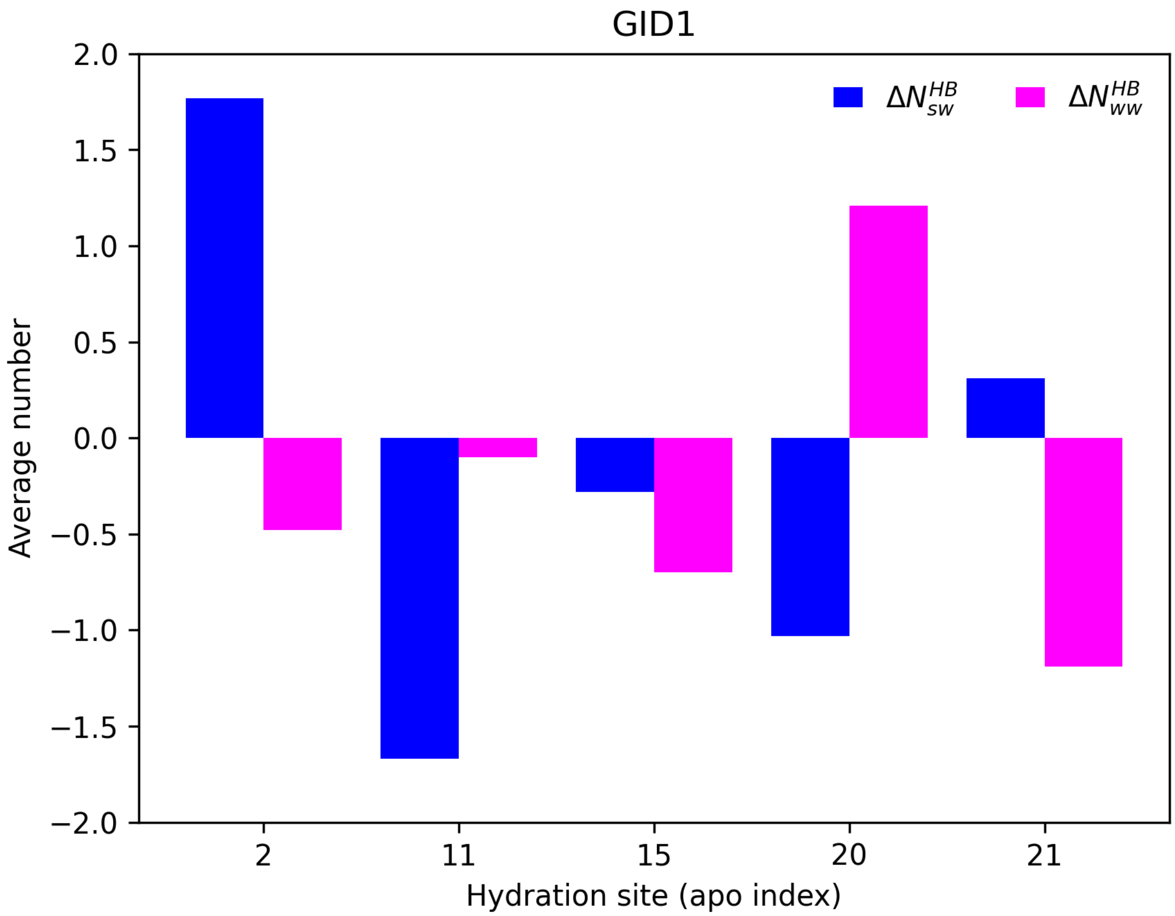

Fig. S21. Difference in hydrogen bonding in conserved *apo* hydration sites and the corresponding *holo* sites in the binding site of GID1 upon ligand binding. N_{sw}^{HB} is the average number of hydrogen bonds between water and solute molecules. N_{ww}^{HB} is the average number of hydrogen bonds between water molecules. Differences were defined between *holo* and *apo* states: $\Delta N_{sw}^{HB} = N_{sw,holo}^{HB} - N_{sw,apo}^{HB}$ and $\Delta N_{ww}^{HB} = N_{ww,holo}^{HB} - N_{ww,apo}^{HB}$.

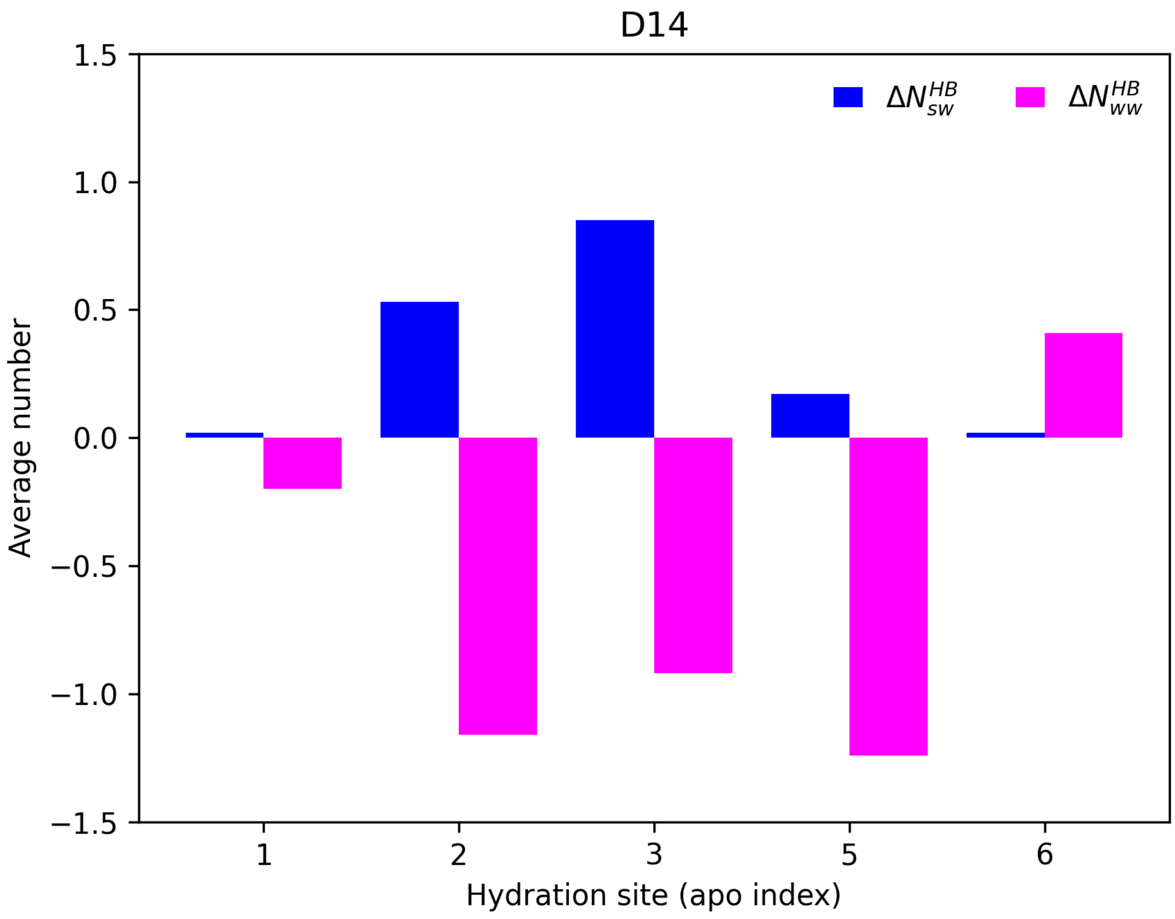

Fig. S22. Difference in hydrogen bonding in conserved *apo* hydration sites and the corresponding *holo* sites in the binding site of D14 upon ligand binding. N_{sw}^{HB} is the average number of hydrogen bonds between water and solute molecules. N_{ww}^{HB} is the average number of hydrogen bonds between water molecules. Differences were defined between *holo* and *apo* states: $\Delta N_{sw}^{HB} = N_{sw,holo}^{HB} - N_{sw,apo}^{HB}$ and $\Delta N_{ww}^{HB} = N_{ww,holo}^{HB} - N_{ww,apo}^{HB}$.

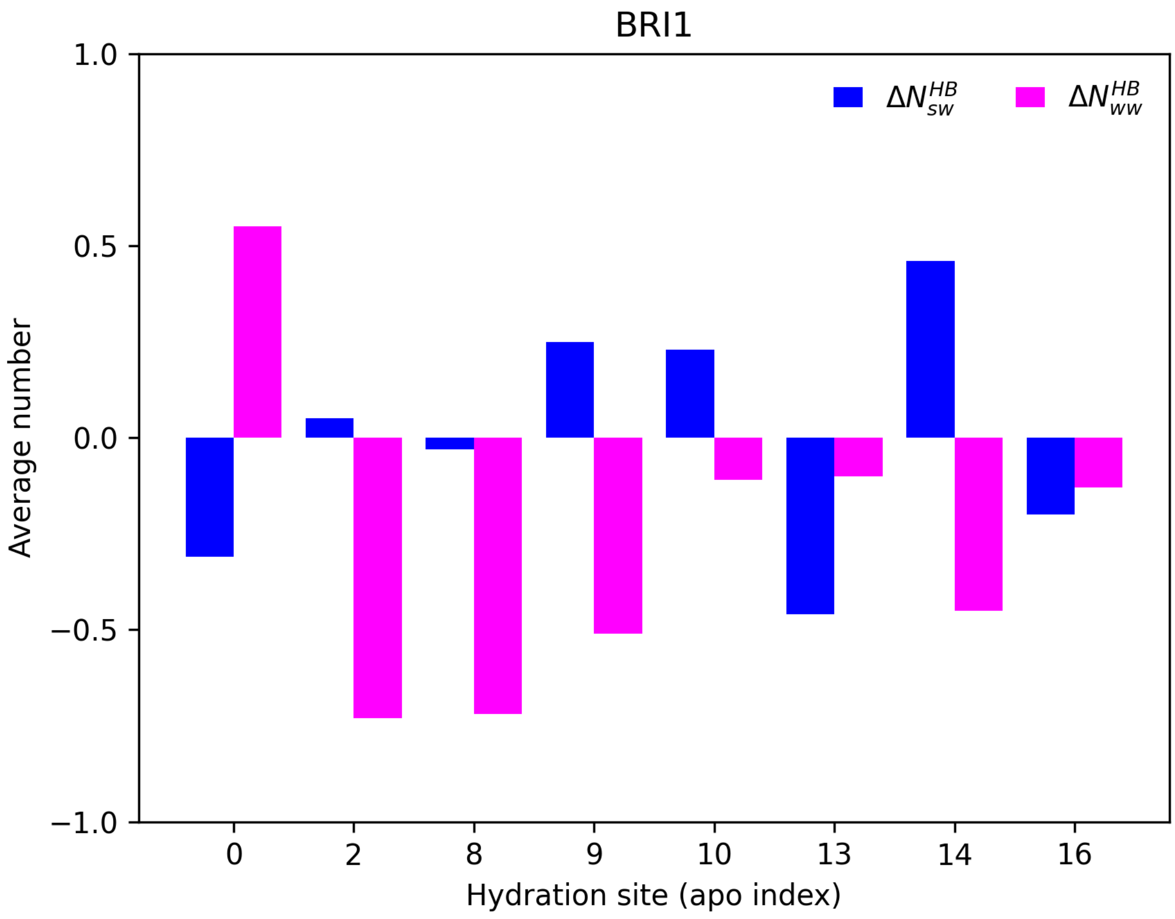

Fig. S23. Difference in hydrogen bonding in conserved *apo* hydration sites and the corresponding *holo* sites in the binding site of BRI1 upon ligand binding. N_{sw}^{HB} is the average number of hydrogen bonds between water and solute molecules. N_{ww}^{HB} is the average number of hydrogen bonds between water molecules. Differences were defined between *holo* and *apo* states: $\Delta N_{sw}^{HB} = N_{sw,holo}^{HB} - N_{sw,apo}^{HB}$ and $\Delta N_{ww}^{HB} = N_{ww,holo}^{HB} - N_{ww,apo}^{HB}$.

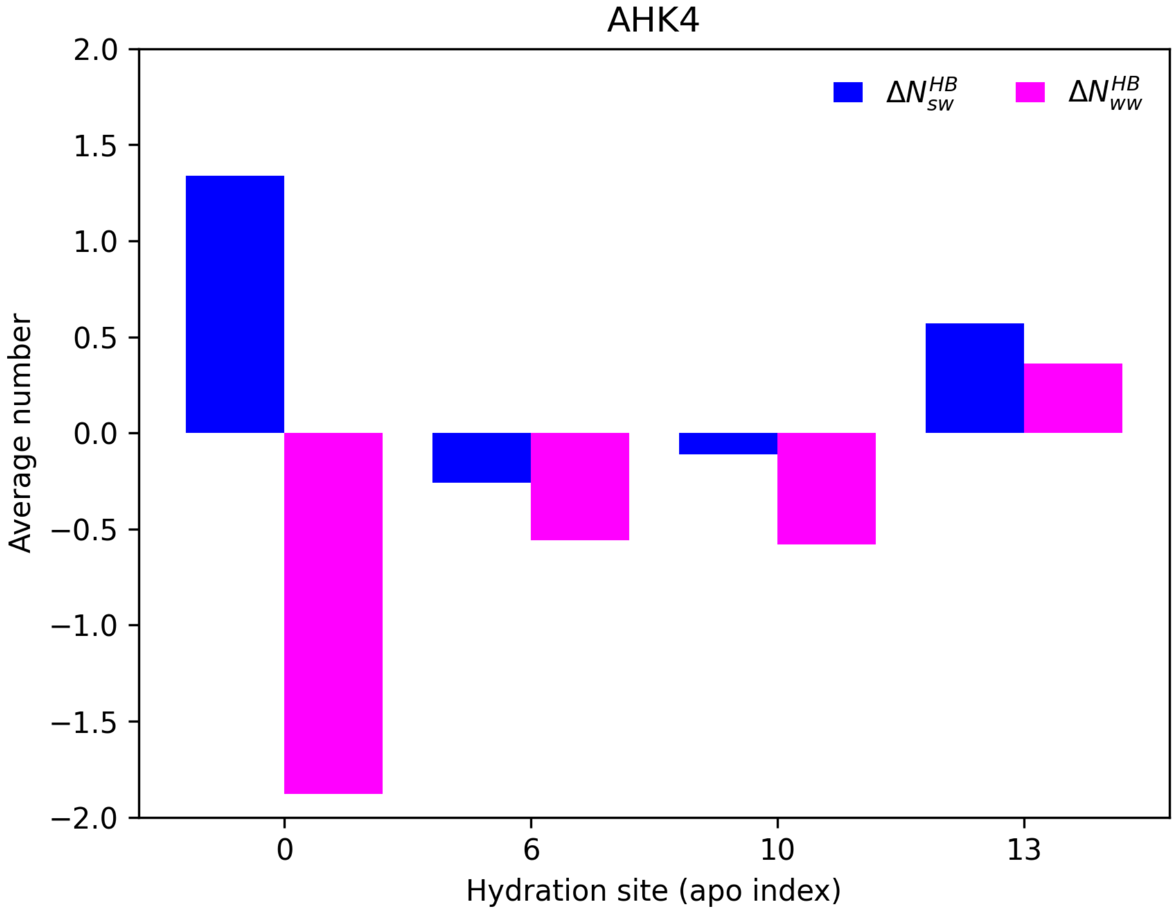

Fig. S24. Difference in hydrogen bonding in conserved *apo* hydration sites and the corresponding *holo* sites in the binding site of AHK4 upon ligand binding. N_{sw}^{HB} is the average number of hydrogen bonds between water and solute molecules. N_{ww}^{HB} is the average number of hydrogen bonds between water molecules. Differences were defined between *holo* and *apo* states: $\Delta N_{sw}^{HB} = N_{sw,holo}^{HB} - N_{sw,apo}^{HB}$ and $\Delta N_{ww}^{HB} = N_{ww,holo}^{HB} - N_{ww,apo}^{HB}$.

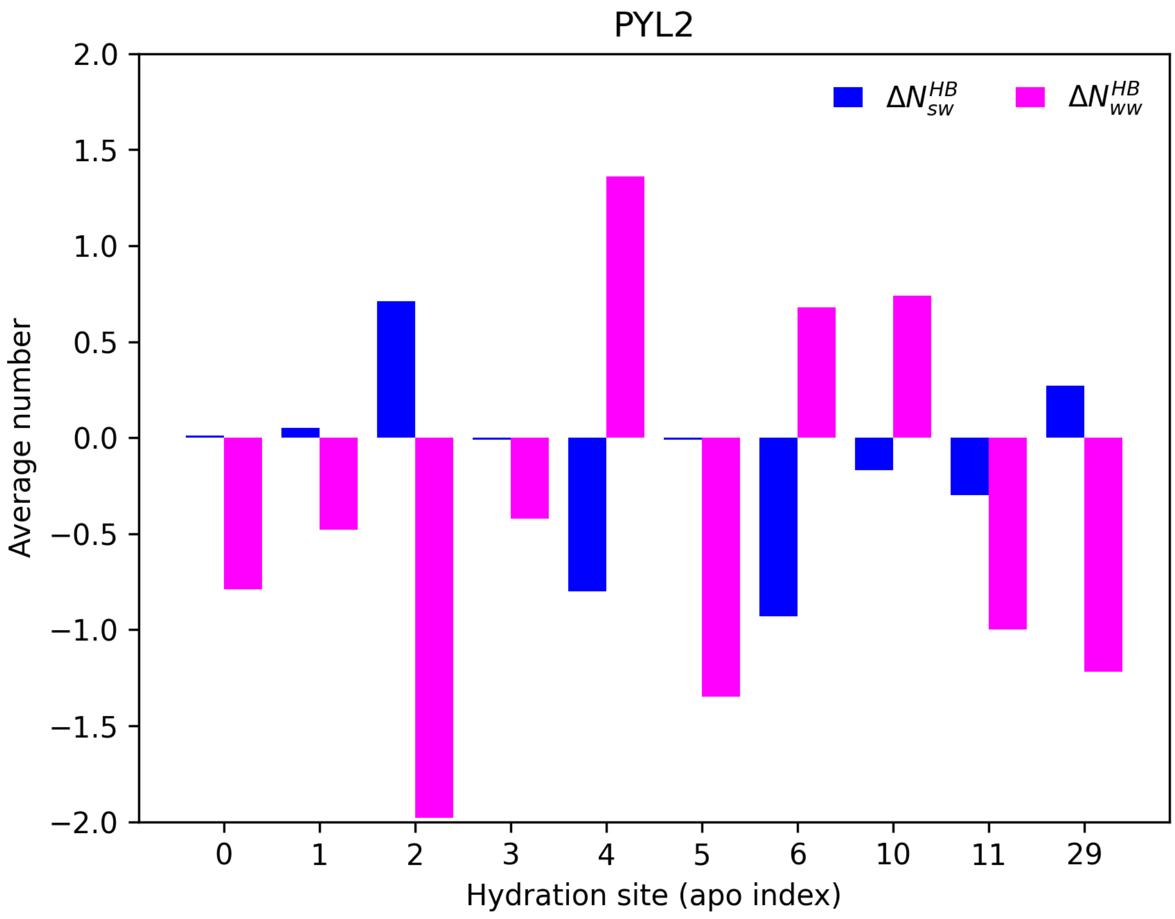

Fig. S25. Difference in hydrogen bonding in conserved *apo* hydration sites and the corresponding *holo* sites in the binding site of PYL2 upon ligand binding. N_{sw}^{HB} is the average number of hydrogen bonds between water and solute molecules. N_{ww}^{HB} is the average number of hydrogen bonds between water molecules. Differences were defined between *holo* and *apo* states: $\Delta N_{sw}^{HB} = N_{sw,holo}^{HB} - N_{sw,apo}^{HB}$ and $\Delta N_{ww}^{HB} = N_{ww,holo}^{HB} - N_{ww,apo}^{HB}$.

11 NPR4 Conformational Change Analysis

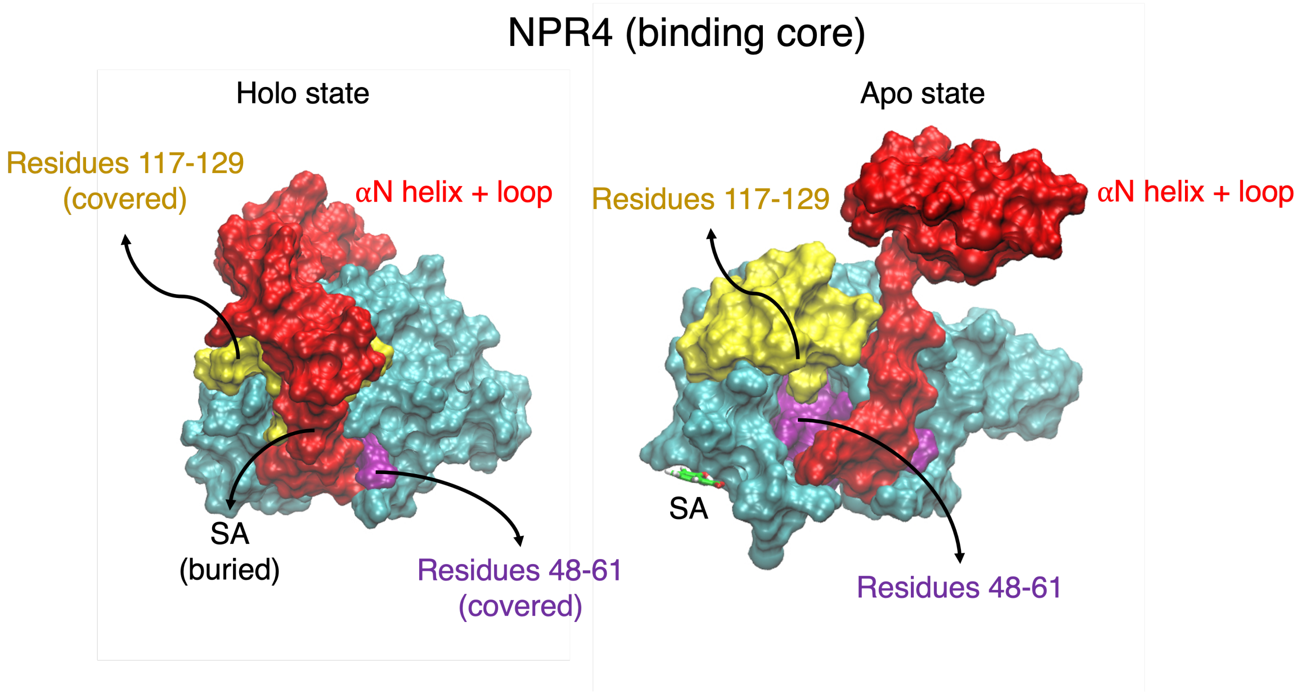

Fig. S26. A conformational change in the αN helix and its connecting loop in the NPR4 salicylic acid binding core explains deuterium exchange profiles for residues 48-61 (412-425 in PDB entry 6WPG) and residues 117-129 (487-499 in PDB entry 6WPG). Experimental data shows that residues 48-61 and 117-129 are protected from solvent in the presence of salicylic acid (SA). Simulations show that the αN helix and its connecting loop shield these regions from solvent, but when the helix moves to release the ligand, they become solvent-exposed. See Extended Data Figure 2 in ref. 13 for experimental data. Also see Figure S8d in this SI.

12 Additional Energy Minima Analysis

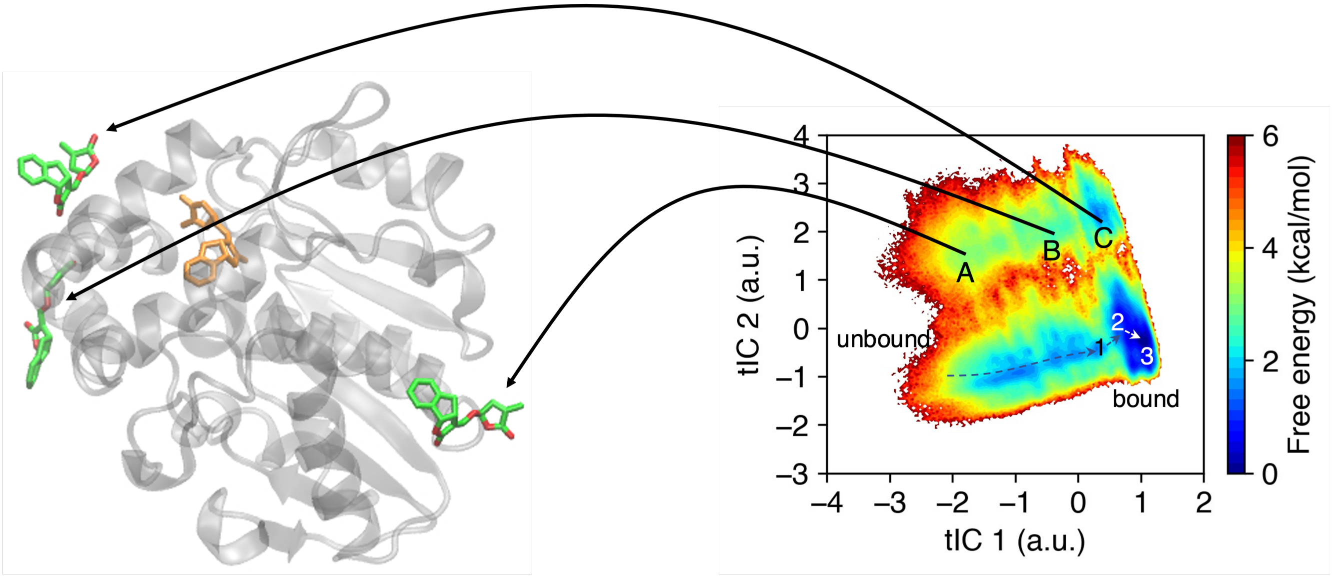

Fig. S27. Additional minima observed in the D14 tIC plot (labeled A-C) are not relevant for binding. These minima correspond to off-pocket interactions, as shown by the locations of the ligand (GR24) on the protein surface (green structures). The correct bound pose for the ligand is shown in orange.

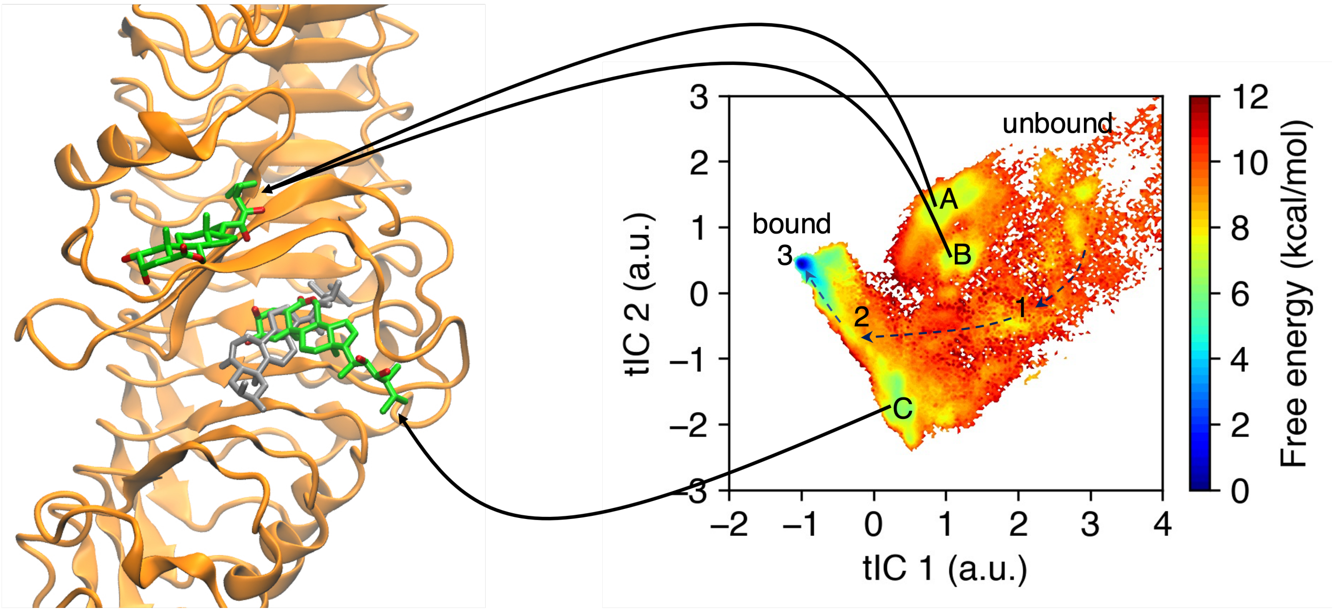

Fig. S28. Additional minima observed in the BRI1 tIC plot (labeled A-C) are not relevant for binding. These minima correspond to off-pocket interactions (A and B) or an incorrect orientation of the ligand in the pocket (C), as shown by the locations of the ligand (brassinolide) on the protein surface (green structures). The correct bound pose for the ligand is shown in gray.
